# ADAP and SKAP55 deficiency suppresses PD-1 expression in CD8^+^ cytotoxic T lymphocytes for enhanced anti-tumor immunotherapy

**DOI:** 10.15252/emmm.201404578

**Published:** 2015-04-07

**Authors:** Chunyang Li, Weiyun Li, Jun Xiao, Shaozhuo Jiao, Fei Teng, Shengjie Xue, Chi Zhang, Chun Sheng, Qibin Leng, Christopher E Rudd, Bin Wei, Hongyan Wang

**Affiliations:** 1 Key Laboratory of Systems Biology, Institute of Biochemistry and Cell Biology, Shanghai Institutes for Biological Sciences, Chinese Academy of SciencesShanghai, China; 2 Shanghai Normal UniversityShanghai, China; 3 Institute Pasteur of Shanghai, Chinese Academy of SciencesShanghai, China; 4 Cambridge Institute of Medical ResearchCambridge, UK; 5 State Key Laboratory of Virology, Wuhan Institute of Virology, Chinese Academy of SciencesWuhan, China

**Keywords:** ADAP/SKAP55, anti-tumor immunotherapy, CsA, CTL cytotoxicity, PD-1

## Abstract

PD-1 negatively regulates CD8^+^ cytotoxic T lymphocytes (CTL) cytotoxicity and anti-tumor immunity. However, it is not fully understood how PD-1 expression on CD8^+^ CTL is regulated during anti-tumor immunotherapy. In this study, we have identified that the ADAP-SKAP55 signaling module reduced CD8^+^ CTL cytotoxicity and enhanced PD-1 expression in a Fyn-, Ca^2+^-, and NFATc1-dependent manner. In DC vaccine-based tumor prevention and therapeutic models, knockout of SKAP55 or ADAP showed a heightened protection from tumor formation or metastases in mice and reduced PD-1 expression in CD8^+^ effector cells. Interestingly, CTLA-4 levels and the percentages of tumor infiltrating CD4^+^Foxp3^+^ Tregs remained unchanged. Furthermore, adoptive transfer of SKAP55-deficient or ADAP-deficient CD8^+^ CTLs significantly blocked tumor growth and increased anti-tumor immunity. Pretreatment of wild-type CD8^+^ CTLs with the NFATc1 inhibitor CsA could also downregulate PD-1 expression and enhance anti-tumor therapeutic efficacy. Together, we propose that targeting the unrecognized ADAP-SKAP55-NFATc1-PD-1 pathway might increase efficacy of anti-tumor immunotherapy.

## Introduction

Cytotoxic T lymphocytes (CTLs) play an important role in anti-tumor responses. Following recognition of tumor antigens on professional dendritic cells (DCs), naive CD8^+^ T cells undergo extensive expansion, acquire effector function, and differentiate into tumor-specific CD8^+^ CTLs to kill tumor cells. Although DC vaccination pulsed with tumor antigens has been shown to prolong the survival time of cancer patients, the majority had only marginal clinical anti-tumor activity (Bhardwaj, 2007). It is critical to dissect the key signaling proteins that determine the efficacy of tumor antigen-induced CD8^+^ CTL cytotoxicity to enhance anti-tumor efficacy.

The inhibitory receptors Programmed Cell Death Receptor 1 (PD-1) and Cytotoxic T-lymphocyte Antigen 4 (CTLA-4, also known as CD152) are inducible and expressed on activated T cells in response to various kinds of antigens such as tumor antigen presented by DCs or tumor cells. In this context, the PD-1 ligand PD-L1 is expressed on a wide variety of tumors, and the blockade of PD-L1 promotes anti-tumor responses (Curiel *et al*, 2003). It has also been demonstrated that the adoptive transfer of PD-1-deficient CD8^+^ TCR transgenic T cells enhances tumor rejection *in vivo* (Blank *et al*, 2004). Therapies targeting PD-1, PD-L1, or CTLA-4 are therefore promising strategies to enhance CTL-mediated anti-tumor efficacy (Gravitz, 2013; Gubin *et al*, 2014; Herbst *et al*, 2014; Powles *et al*, 2014; Tumeh *et al*, 2014). Despite this, it has not been fully delineated how to prevent PD-1 or CTLA-4 expression in T cells during cancer immunotherapy.

T-cell activation is mediated by a protein-tyrosine phosphorylation cascade including Lck, Fyn, ZAP-70 and their phosphorylation of immune cell-specific adaptor proteins. These adaptor proteins lack definable catalytic activities, but instead, possess binding domains or sites for the formation of multimeric complexes. In this study, we have identified that adaptors SKAP55 (src kinase-associated protein of 55 kDa; also termed SKAP1/src kinase-associated protein-1) and ADAP (adhesion and degranulation promoting adaptor protein; also known as Fyb/Fyn binding protein; or SLAP-130/SLP-76-associated protein of 130 kDa) are located at the killing synapses between CD8^+^ CTLs and tumor cells. Previous studies have shown that SKAP55 and ADAP are expressed predominantly in T cells, and constitutively bind together (Liu *et al*, 1998; Marie-Cardine *et al*, 1998). Further, ADAP could stabilize SKAP55 expression at protein level (Huang *et al*, 2005; Wang *et al*, 2007). ADAP also interacts with SLP-76 (Src homology 2 (SH2) domain-containing leukocyte protein of 76 kDa), and SKAP55 binds RapL (regulator for cell adhesion and polarization enriched in lymphoid tissues) or RIAM (Rap1-GTP-interacting adaptor molecule) to generate the ‘inside-out’ signaling pathway for integrin activation and T-cell adhesion (Griffiths *et al*, 2001; Peterson *et al*, 2001; Wang *et al*, 2003, 2004, 2007; Menasche *et al*, 2007; Raab *et al*, 2010). Since integrin-mediated killing synapse formation facilitates CD8^+^ CTLs to lyse tumor cells (Schmits *et al*, 1996; Franciszkiewicz *et al*, 2013), we expected that ADAP and SKAP55 might enhance CD8^+^ CTL cytotoxicity. Surprisingly, this study shows that deficiency of ADAP or SKAP55 enhances CD8^+^ CTL cytotoxicity and increases the efficacy of tumor prevention and therapy *in vivo*. Interestingly, their deficiency specifically suppresses PD-1 expression in CD8^+^ effector T cells without affecting CTLA-4 expression or the percentages of tumor-infiltrating CD4^+^Foxp3^+^ regulatory T cells (Tregs). We suggest the ADAP-SKAP55-NFATc1-PD-1 axis as a novel mechanism to improve CD8^+^ CTL-mediated anti-tumor response.

## Results

### SKAP55 decreases CD8^+^ CTL cytotoxicity with enhanced PD-1 expression

Previous studies have demonstrated that SKAP55 and ADAP positively regulate integrin-mediated T-cell adhesion (Griffiths *et al*, 2001; Peterson *et al*, 2001; Wang *et al*, 2003, 2007). Integrin plays a critical role in synapse formation between CD8^+^ CTLs and tumor cells to enhance cytotoxicity (Schmits *et al*, 1996; Franciszkiewicz *et al*, 2013). We therefore asked whether SKAP55 or ADAP could increase CD8^+^ CTL cytotoxicity.

**Figure 1:**
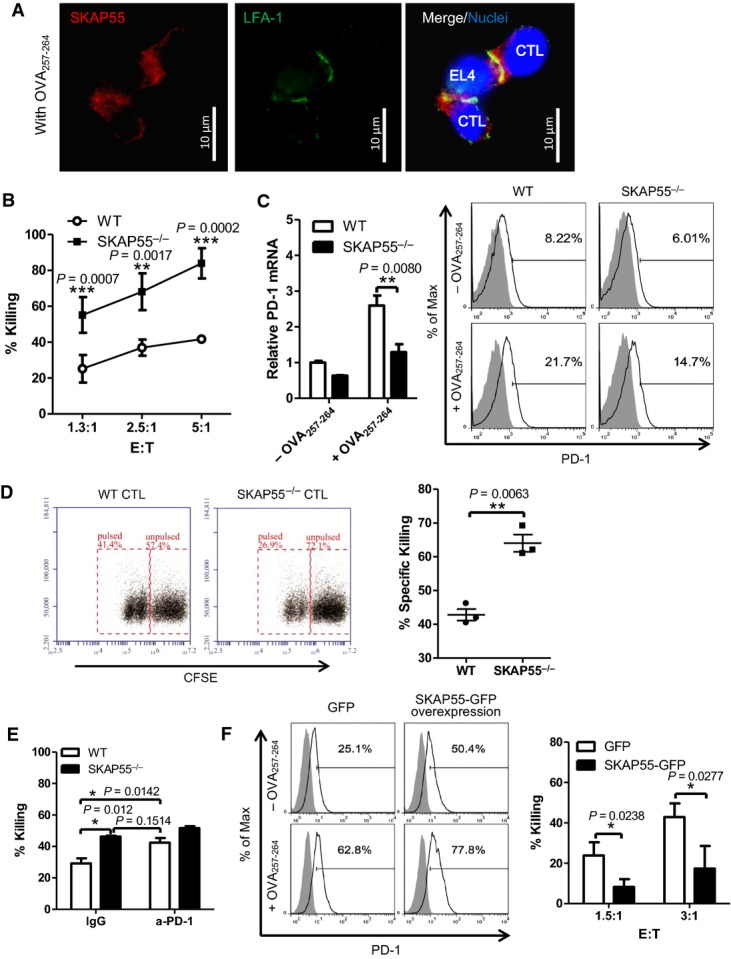
SKAP55 enhances PD-1 expression to decrease CD8^+^ CTL cytotoxicity

Wild-type or SKAP55 knockout (KO) mice were crossed with OT-I mice, and naïve OT-I CD8^+^ T cells were isolated and incubated with 10 nM OVA_257-264_ peptide-pulsed splenocytes to generate OVA_257-264_-specific CD8^+^ CTLs. To examine *in vitro* cytotoxicity, wild-type or SKAP55 KO OT-I CD8^+^ CTLs were incubated with 10 nM OVA_257-264_-pulsed EL-4 cells, which were derived from lymphoma of C57Bl6 mice and used as tumor targets. As expected, SKAP55 was recruited together with LFA-1 to the killing synapse between OT-I CD8^+^ CTLs and the tumor EL-4 cells (Fig1A). During the priming phase, we observed that SKAP55-deficient CD8^+^ cells reduced IL-2 production (Supplementary Fig S1A) but did not affect LFA-1 (i.e. CD11a) expression (Supplementary Fig S1B).

A OT-I CD8^+^ CTLs were conjugated with 10 nM OVA_257-264_-pulsed EL-4 cells for 30 min, fixed, and stained with anti-SKAP55 (red), anti-LFA-1 (green) and Hoechst (blue).

B, C WT and SKAP55^−/−^ OT-I CD8^+^ CTLs were generated from WT or SKAP55^−/−^ OT-I Tg mice, then incubated with 10 nM OVA_257-264_-pulsed EL4 targets for 4 h to assess the *in vitro* cytotoxicity at different Effector:Target ratios (B; mean of triplicates ± SD); surface expression and the mRNA levels of PD-1 (C). Graphs are representative of at least three independent experiments.

D WT and SKAP55^−/−^ OT-I CD8^+^ CTLs (3 × 10^6^) were injected into C57BL/6 mice, followed by injection of non-pulsed (CFSE^hi^) or 10 nM OVA_257-264_-pulsed (CFSE^lo^) splenocytes (5 × 10^6^) to measure *in vivo* cytotoxicity (mean ± SD, *n* = 3 mice per group). Representative data of three independent experiments.

E WT and SKAP55^−/−^ OT-I CD8^+^ CTLs were pretreated with anti-PD-1 antibody or IgG control, followed by incubation with OVA_257-264_-pulsed EL4 cells to examine *in vitro* killing ability (mean of triplicates ± SD). Graphs are representative of three independent experiments.

F CD8^+^ CTLs were transfected with plasmids expressing SKAP55-GFP or EGFP, then treated with non-pulsed or 10 nM OVA_257-264_-pulsed EL4 cells to examine surface PD-1 expression (left panel) or the *in vitro* killing assay (mean of triplicates ± SD) (right panel).

Data information: Statistical significance was determined with unpaired two-tailed Student's *t*-test. Graphs are representative of three independent experiments.

Surprisingly, SKAP55 KO OT-I CD8^+^ CTLs showed greater cytotoxicity against OVA_257-264_-pulsed EL-4 cells *in vitro* compared with wild-type controls at various effector-to-target ratios (Fig1B). Interestingly, the absence of SKAP55 also decreased PD-1 expression both at mRNA and cell surface levels in OT-I CD8^+^ CTLs (Fig1C). In naïve or resting wild-type or SKAP55 KO CD8^+^ T cells, PD-1 was expressed at basal levels without significant difference.

Next, we used an *in vivo* method to assess the role of SKAP55 to kill targets *in vivo*. Wild-type or SKAP55 KO OT-I CD8^+^ CTLs were i.v. injected into the recipient mice; then OVA_257-264_-pulsed splenocytes (i.e. CFSE^low^) and non-pulsed splenocytes (i.e. CFSE^high^) were used as target cells and i.v. injected together at 1:1 ratio into the recipient mice. Non-pulsed splenocytes were not lysed and used as internal controls. Compared to the wild-type controls, SKAP55 KO OT-I CD8^+^ CTLs killed more OVA_257-264_-pulsed splenocytes *in vivo*, and flow cytometry data showed that 26.9% vs. 41.5% alive CFSE^low^-target cells left after the injection of SKAP55 KO or wild-type CTLs) (Fig1D). Importantly, the addition of anti-PD-1 antibody to wild-type OT-I CTLs restored their killing ability *in vitro* to a similar level as that of SKAP55 KO CTLs (Fig1E).

We then over-transfected GFP-SKAP55 into OT-I CD8^+^ CTLs (Supplementary Fig S1C), and GFP-SKAP55^+^ cells were used in a cytotoxicity assay. Overexpression of SKAP55 enhanced surface PD-1 levels and reduced CD8^+^ CTL cytotoxicity compared to GFP^+^ control cells (Fig1F). By using the genetic deficiency and overexpression strategy, we demonstrated that SKAP55 unexpectedly inhibits CD8^+^ CTL cytotoxicity with enhanced PD-1 expression.

### ADAP-deficient CD8^+^ CTLs reduce PD-1 expression and enhance cytotoxicity

**Figure 2:**
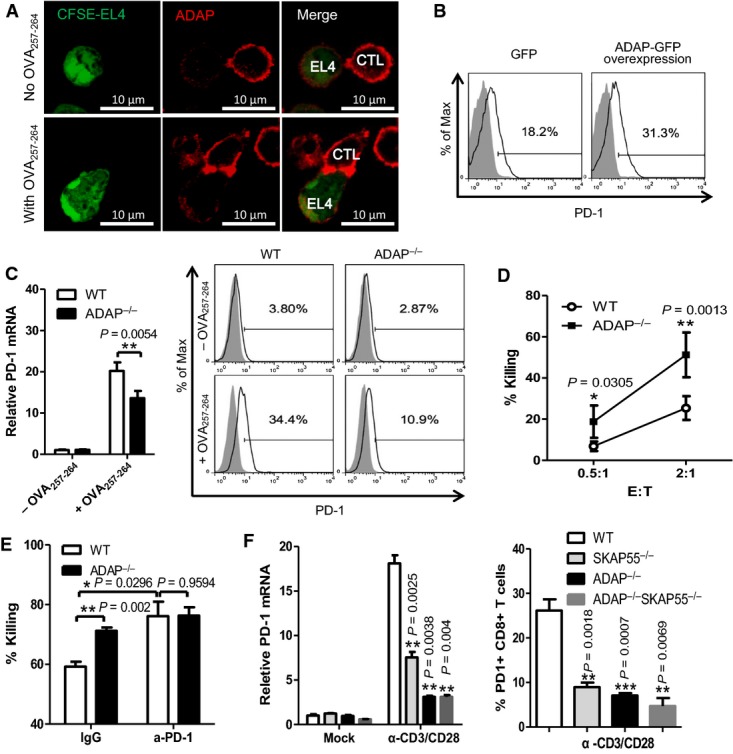
ADAP-deficient CD8^+^ CTLs reduce PD-1 expression and enhance cytotoxicity

Because ADAP was reported to bind and stabilize SKAP55 at the protein level (Huang *et al*, 2005), we next checked the role of ADAP in regulating PD-1 expression and cytotoxic function of CD8^+^ CTL. After priming with the OVA_257-264_ peptide, ADAP-deficient CD8^+^ CTLs reduced IL-2 production without changing LFA-1 expression (Supplementary Fig S2A and B). Similar to SKAP55, ADAP accumulated at the killing synapse between OT-I CD8^+^ CTLs and OVA_257-264_-pulsed EL-4 cells (Fig2A). Overexpression of ADAP in CD8^+^ CTLs (Supplementary Fig S2C) also enhanced surface PD-1 expression compared to the control CTLs (Fig2B), while ADAP KO OT-I CTLs decreased PD-1 expression at the protein and mRNA levels (Fig2C). In agreement with the reduced PD-1 expression levels, ADAP-deficient OT-I CD8^+^ CTLs enhanced killing against OVA_257-264_-pulsed EL-4 cells at various E:T ratios *in vitro* (Fig2D). Significantly, anti-PD-1 antibody treatment could increase the *in vitro* killing ability of wild-type OT-I CTLs to a similar level as that of ADAP^−/−^ CTLs (Fig2E).

A OT-I CD8^+^ CTLs were conjugated with CFSE-labeled OVA_257-264_-pulsed or non-pulsed EL-4 cells for 30 min, fixed, and stained with anti-ADAP (red).

B OT-I CD8^+^ CTLs were transfected with plasmids expressing ADAP or GFP, then stimulated with 10 nM OVA_257-264_-pulsed EL-4 cells to detect surface PD-1 expression. Representative of three independent experiments.

C, D WT and ADAP^−/−^ OT-I CD8^+^ CTLs were stimulated with 10 nM OVA_257-264_-pulsed or unpulsed EL-4 cells for 4 h to examine surface expression and the mRNA levels of PD-1 (C), *in vitro* cytotoxicity at different Effector:Target ratios (D; mean of triplicates ± SD). Graphs are representative of at least three independent experiments.

E WT and ADAP^−/−^ OT-I CD8^+^ CTLs were pretreated with 10 μg/ml anti-PD-1 antibody or IgG control, followed by incubation with 10 nM OVA_257-264_-pulsed EL4 cells to examine *in vitro* killing ability (mean of triplicates ± SD).

F Naïve CD8^+^ T cells were isolated from WT, SKAP55^−/−^, ADAP^−/−^, and SKAP55^−/−^ADAP^−/−^ splenocytes, then stimulated with plate-bound anti-CD3/CD28 for 12 h to assess PD-1 mRNA levels, and for 48 h to check surface PD-1 expression (mean of triplicates ± SD).

Data information: Statistical significance was determined with unpaired two-tailed Student's *t*-test. Graphs are representative of three independent experiments.

Previous studies have demonstrated that ADAP could stabilize SKAP55 expression at protein level (Huang *et al*, 2005; Wang *et al*, 2007). To examine the effect of ADAP/SKAP55 double KO (DKO), we crossbred ADAP KO mice with SKAP55 KO mice to get DKO mice. After stimulated with plate-bound anti-CD3/CD28, DKO CD8^+^ T cells decreased PD-1 expression at protein and mRNA levels as those in ADAP KO CD8^+^ T cells (Fig2F). Also, anti-CD3/CD28-stimulated DKO CD8^+^ T cells reduced IL-2 mRNA levels to similar degree as that in ADAP KO CD8^+^ T cells (Fig S2D). Taken together, we suggest that the ADAP-SKAP55 module reduces CD8^+^ CTL cytotoxicity and enhances PD-1 expression.

### The ADAP-SKAP55 module is dependent on NFATc1, Ca^2+^, and Fyn to control PD-1 expression

**Figure 3:**
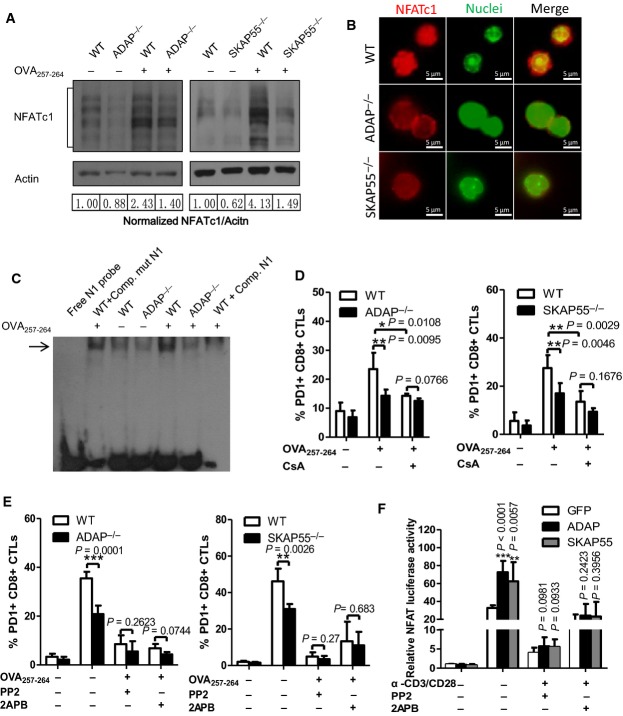
The ADAP-SKAP55 module is dependent on NFATc1, Ca^2+^, and Fyn to control PD1 expression

The transcription factors NFATc1 (nuclear factor of activated T cells c1, also known as NFAT2) (Agnellini *et al*, 2007; Oestreich *et al*, 2008), c-Fos (Xiao *et al*, 2012) and Blimp-1 (Shin *et al*, 2009) have been reported to enhance PD-1 expression. We next asked which transcription factor cooperated with SKAP55 or ADAP to regulate PD-1 expression in CD8^+^ CTLs. According to previous findings, activated NFATc1 is highly dephosphorylated and moves faster in SDS–PAGE gels than the fully phosphorylated inactive form. We noticed that OVA_257-264_ peptide stimulation elevated the protein expression levels of total and activated NFATc1 in wild-type CD8^+^ CTLs, which were significantly suppressed in ADAP- or SKAP55-deficient CD8^+^ CTLs (Fig3A). The immunostaining data confirmed that the expression levels of total NFATc1 and the nuclear localized NFATc1 were reduced in OVA_257-264_ peptide-stimulated ADAP KO as well as SKAP55 KO CD8^+^ CTLs (Fig3B, quantified data in Supplementary Fig S3A). In addition, similar to ADAP KO CD8^+^ T cells, the plate-bound anti-CD3/CD28-stimulated DKO CD8^+^ T cells also reduced NFATc1 mRNA levels (Supplementary Fig S3B). We next measured the interaction of NFATc1 with the PD-1 promoter by an electrophoretic mobility shift assay (EMSA) with the biotin-labeled NFAT-binding DNA probes (N1). In response to OVA_257-264_ peptide stimulation, we observed the enhanced amount of the DNA probe/protein complexes in wild-type CD8^+^ CTLs, while this enhancement was inhibited in ADAP-deficient or SKAP55-deficient CD8^+^ CTLs (Fig3C and Supplementary Fig S3C). We used the unlabeled competitor probe (Comp. N1) and the mutant probe that contains the same sequence except for carrying a mutated NFAT-binding site (Comp. mutN1) as controls. Comp. N1 abrogated the formation of the probe-NFAT complex, while Comp. mutN1 failed to achieve this effect. It indicates specificity of the NFAT-binding probe, which was also described by previous studies (Oestreich *et al*, 2008). In response to OVA_257-264_ peptide stimulation, we observed the enhanced amount of the DNA probe/protein complexes in wild-type CD8^+^ CTLs, while this enhancement was inhibited in ADAP-deficient or SKAP55-deficient CD8^+^ CTLs (Fig3C and Supplementary Fig S3C).

A–C WT, ADAP^−/−^, or SKAP55^−/−^ OT-I CD8^+^ CTLs were stimulated with OVA_257-264_-pulsed or unpulsed EL4 cells to detect the levels of NFATc1 expression, activation and nuclear entry by immunoblotting (A) or immunostaining (B). Alternatively, nuclear extracts from these CTLs were incubated with the N1 DNA probes containing NFAT-binding sites for an EMSA. The unlabeled competitor probe (Comp. N1) and the mutant probe that contains the same sequence except for carrying a mutated NFAT-binding site (Comp. mutN1) were included as controls (C).

D WT, ADAP^−/−^, or SKAP55^−/−^ OT-I CD8^+^ CTLs were pretreated with DMSO or 5 μM CsA, then stimulated with OVA_257-264_-pulsed EL-4 cells for 4 h to examine surface PD-1 expression (mean of triplicates ± SD).

E WT, ADAP^−/−^, or SKAP55^−/−^ OT-I CTLs were pretreated with the DMSO control, 10 μM PP2 or 75 μM 2APB, then stimulated with OVA_257-264_-pulsed EL-4 cells for 4 h to examine surface PD-1 expression (mean of triplicates ± SD).

F GFP, ADAP, or SKAP55 was overexpressed with pGL3-NFAT Luciferase reporter plasmid into Jurkat cells. Cells were stimulated with anti-CD3 and anti-CD28 in the presence or absence of PP2 or 2APB for 6 h, followed by measuring luciferase readings (mean of triplicates ± SD).

Data information: Statistical significance was determined with unpaired two-tailed Student's *t*-test. Graphs are representative of three independent experiments. Source data are available online for this figure.

Previous report suggested that c-Fos also enhances PD-1 expression to a much lesser degree compared to NFATc1 (Oestreich *et al*, 2008). We observed that ADAP KO or SKAP55 KO CD8^+^ CTLs reduced the mRNA levels of c-Fos (Supplementary Fig S3D), but did not affect Blimp1 expression (Supplementary Fig S3E). The inhibitor cyclosporine A (CsA) was reported to block NFATc1 expression and nuclear translocation (Flanagan *et al*, 1991), but increase mRNA levels and the nuclear translocation of c-Fos (Su *et al*, 1996; Navarro-Antolin *et al*, 2000). Interestingly, our data showed that CsA treatment significantly decreased the mRNA levels and surface expression of PD-1 in wild-type CD8^+^ CTLs (Supplementary Fig S3F). This indicates that reduced NFATc1 following CsA treatment plays a dominant role in the reduction of PD-1 expression (even under the condition that CsA treatment enhances c-Fos expression). Furthermore, CsA treatment reduced PD1 expression in wild-type CD8^+^ CTLs to similar levels as those in ADAP KO or SKAP55 KO CD8^+^ CTLs (Fig3D). Together, we have demonstrated that ADAP and SKAP55 increase antigen-induced NFATc1 expression and activity in CD8^+^ CTLs.

We next dissected which intracellular signaling effectors cooperated with ADAP or SKAP55 to determine the NFATc1-driven PD-1 expression. We and others have previously identified that the tyrosine kinase Fyn phosphorylates and interacts with ADAP and SKAP55 after TCR stimulation (Marie-Cardine *et al*, 1997, 1998; Liu *et al*, 1998). Fyn also physically interacts with and activates SHP-2 (Tang *et al*, 1999), while PD-1 recruits and transduces negative signals mainly via SHP-2 (Yokosuka *et al*, 2012). We performed an immunoprecipitation assay with anti-Fyn, which pulled down endogenous SKAP55 or ADAP, and vice versa. Interestingly, anti-SHP-2 or anti-PD-1 co-precipitated endogenous Fyn, SKAP55 or ADAP in CD8^+^ CTLs (Supplementary Fig S3G). We then overexpressed SKAP55 (or ADAP) together with PD-1 or SHP-2 in 293 cells, but did not observe a direct interaction.

In agreement with the negative role of PD-1, Fyn deficiency enhances CD8^+^ T-cell effector function (Filby *et al*, 2007). Further, anti-CD3-stimulated Fyn-deficient T cells severely diminished dephosphorylation and nuclear translocation of NFAT as well as calcium flux (Sugie *et al*, 2004), while calcium signaling was reported to induce NFAT nuclear translocation (Macian, 2005). We therefore examined whether the ADAP-SKAP55 module was dependent on calcium and Fyn kinase activity to induce PD-1 expression. After treatment with the specific inhibitor 2APB (2-aminoethoxydiphenyl borate) or PP2 to block calcium signaling or Fyn activity respectively, wild-type, ADAP KO or SKAP55 KO CD8^+^ CTLs significantly decreased surface PD-1 expression to similar levels (Fig3E).

Next, we measured NFAT activity by overexpressing ADAP or SKAP55 in Jurkat cells together with the NFAT-luciferase reporter plasmid, which contains three tandem repeats of the NFAT-binding site. After anti-CD3 and anti-CD28 stimulation, overexpression of ADAP or SKAP55 enhanced NFAT luciferase readings, while PP2 and 2APB blocked this to the same levels as wild-type cells (Fig3F). In addition, deletion of the SH3 domain of SKAP55 (termed SKAP55-ΔSH3), which disrupts the ADAP-SKAP55 interaction (Duke-Cohan *et al*, 2006), could abolish the enhancement of NFAT luciferase activity (Supplementary Fig S3H). Previous reports suggest that activated NFATc1 binds the *IL-2* promoter to upregulate IL-2 expression, and exposure of CD8^+^ T cells to high IL-2 hinders the generation of functional memory CD8^+^ effector cells with enhanced PD-1 expression (de Goër de Herve *et al*, 2012). This is consistent with our observation that SKAP55 deficiency or ADAP deficiency reduced PD-1 expression, IL-2 production, and NFAT activation to enhance cytotoxicity of CD8^+^ CTLs.

### SKAP55-deficient mice enhance DC-based vaccine for prevention of tumors *in vivo*

We next asked whether SKAP55 or ADAP deficiency enhanced the DC vaccine-induced prevention of tumors *in vivo*. Wild-type, SKAP55^−/−^ or ADAP^−/−^ mice were immunized twice with OVA_257-264_-pulsed DCs, then s.c. injected with E.G7 lymphoma cells. E.G7 cells are generated based on EL4 cells, which endogenously synthesizes and presents OVA_257-264_ peptide. SKAP55^−/−^ mice or ADAP^−/−^ mice significantly repressed tumor formation under the skin compared to wild-type mice (Supplementary Fig S4A).

**Figure 4:**
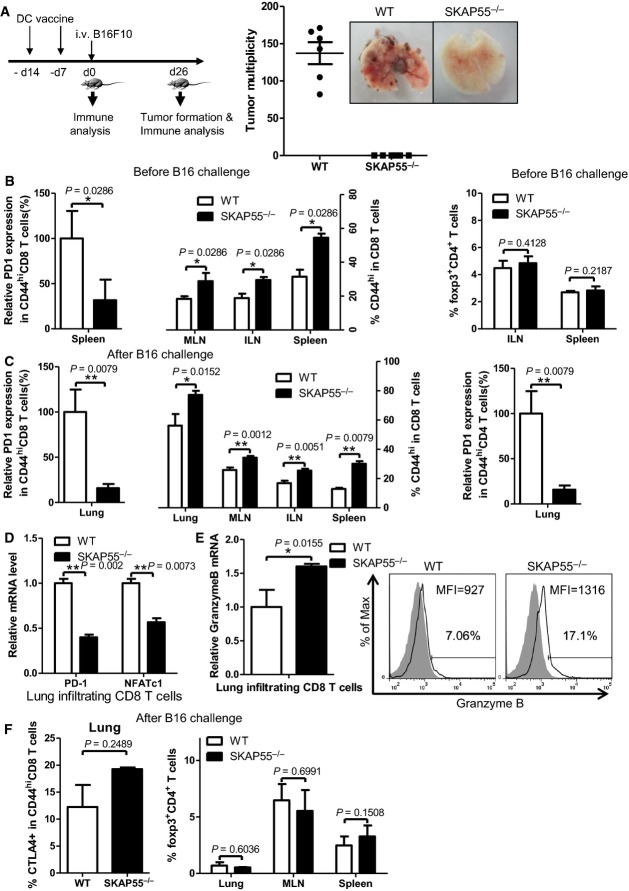
SKAP55-deficient mice enhance DC-based vaccine for prevention of tumors *in vivo*

To confirm this phenotype in E.G7 (or EL4) cells is neither cell-line nor tumor-type specific, we next used DC vaccine-mediated tumor prevention model under physiological conditions. DCs were pulsed with the melanoma B16F10 tumor lysates and s.c. injected twice to wild-type or SKAP55^−/−^ mice. These mice were then challenged i.v. with B16F10 cells, and tumor formation was examined in lungs 26 days later (Fig4A, left panel). Multiple tumors formed in lungs from wild-type mice, but the immunized SKAP55^−/−^ mice strongly inhibited tumor formation (Fig4A, right panel).

A WT and SKAP55 KO C57BL/6 mice were s.c. immunized on day −14 and day −7 by DCs that were prepulsed with B16F10 tumor lysates. On day 0, the immunized mice were i.v. inoculated with B16F10 cells. Number of lung tumors were counted 26 days after B16F10 challenge (*n* ≥ 5 mice).

B, C At day 0 (B, before tumor injection) or at day 26 (C, after tumor injection), surface PD-1 expression in CD44^hi^CD8^+^ or CD44^hi^CD4^+^ T cells, and the percentages of CD44^hi^CD8^+^ T cells or Foxp3^+^CD4^+^ cells were analyzed (mean ± SD, *n* ≥ 5 mice).

D, E The mRNA levels of PD-1, NFATc1, or the mRNA and protein levels of granzyme B in lung infiltrating CD8^+^ T cells were examined at day 26 (mean of triplicates ± SD, and the mRNA samples were prepared from at least 3 mice per group).

F CTLA-4 levels in the lung-infiltrated CD44^hi^CD8^+^ T cells or the percentages of CD4^+^Foxp3^+^ T cells in lungs, MLN, and spleen were measured at day 26 (mean ± SD, *n* ≥ 5 mice).

Data information: Statistical significance was determined with Mann–Whitney *U*-test. Data are representative of three independent experiments.

We then characterized T-cell populations at different time points post-DC vaccination. According to previous studies, CD44^hi^CD8^+^ T cells are potent cytolytic killers with increased levels of CD25, perforin, and granzyme B, which could differentiate into CD8^+^ central memory-like T cells (Zhang *et al*, 2004). We found that 7 days after the second DC immunization (i.e. day 0, without tumor challenge), the percentages of CD44^hi^CD8^+^ effector/memory T cells were enhanced in spleens, and mesentery or inguinal lymph nodes (MLNs or ILNs) from the immunized SKAP55^−/−^ mice, while their surface PD-1 expression was significantly decreased (Fig4B, left panel). Interestingly, the percentages of CD4^+^Foxp3^+^T regulatory cells (Tregs) in spleens and lymph nodes remained unchanged in the immunized SKAP55-deficient mice (Fig4B, right panel).

After the immunized SKAP55 KO mice were challenged with the B16F10 tumor cells for 26 days, the percentages of CD44^hi^CD8^+^ effector/memory T cells in lungs, spleens, ILNs, MLNs were still kept at higher levels (Fig4C, left panel). Importantly, the lung tumor infiltrating SKAP55 KO CD44^hi^CD8^+^ (Fig4C, left panel) or CD44^hi^CD4^+^ (Fig4C, right panel) T cells significantly reduced surface PD-1 expression. Consistent with this, the mRNA levels of PD-1 and NFATc1 (Fig4D) were reduced, while the mRNA or protein levels of granzyme B (Fig4E) and IFN-γ (Supplementary Fig S4B) were enhanced in lung infiltrating CD8^+^ T cells from the SKAP55 KO mice. However, SKAP55 deficiency did not substantially affect the expression levels of perforin, CD25 and CD69 (Supplementary Fig S4B). Next, we assessed the percentages of KLRG1^hi^CD127^low^ CD8^+^ T cells in lungs, which represent a population of terminally differentiated short-lived effector T cells. In consistent with the enhanced anti-tumor efficacy, the immunized SKAP55 KO mice increased the percentages of infiltrating KLRG1^hi^CD127^low^ CD8^+^ T cells in lungs (Supplementary Fig S4C).

Interestingly, even after tumor challenge, the expression levels of total CTLA-4 in CD44^hi^CD8^+^ T cells, and the percentages of CD4^+^Foxp3^+^ Tregs in lung tumors, spleens, or lymph nodes were not reduced in the immunized SKAP55^−/−^ mice (Fig4F). We also observed that the percentages of antigen-presenting cells including CD11c^+^ DCs or CD11b^+^F4/80^+^ macrophages were normal in spleens or draining LN from the immunized SKAP55 KO mice (Supplementary Fig S4D).

### ADAP-deficient mice enhance DC-based vaccine for the prevention of tumors *in vivo*

**Figure 5:**
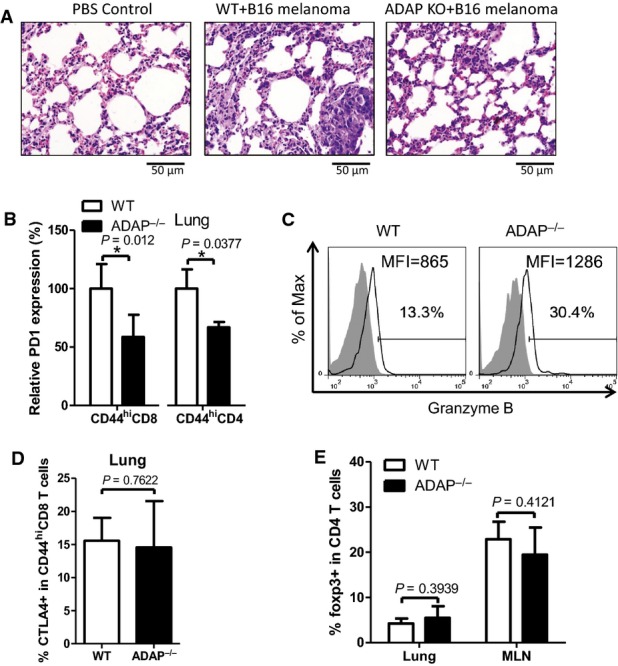
ADAP-deficient mice enhance DC-based vaccine for prevention of tumors *in vivo*

In agreement with the finding that ADAP stabilizes SKAP55 at protein levels (Huang *et al*, 2005), we observed a similar role for ADAP in DC vaccine-induced anti-tumor response *in vivo*. After being immunized twice with the B16F10 tumor lysate-pulsed DCs, ADAP^−/−^ mice significantly repressed tumor formation in lungs (Supplementary Fig S5). HE staining revealed tumor formation in the lungs of wild-type mice, which was barely found in lungs of the ADAP KO mice (Fig5A). By analyzing lung tumor infiltrating T cells, we observed the enhanced populations of KLRG1^hi^CD127^low^ effector CD8^+^ T cells in the immunized ADAP KO mice (Supplementary Fig S4C). Further, ADAP deficiency decreased surface PD-1 expression in CD44^hi^CD8^+^ T cells as well as in CD44^hi^CD4^+^ T cells (Fig5B). The increased granzyme B levels were also observed in ADAP KO CD8^+^ T cells (Fig5C). In contrast, ADAP deficiency did not affect total CTLA-4 expression in CD44^hi^CD8^+^ T cells (Fig5D), or the percentages of CD4^+^FoxP3^+^ Tregs in lungs or MLN (Fig5E). Collectively, the increased population of CD44^hi^CD8^+^ T cells in DC-immunized SKAP55^−/−^ mice or ADAP^−/−^ mice, which decreased PD-1 expression (but not CTLA-4) and increased granzyme B levels, might enhance the protection against tumor formation.

A The same procedures as in Fig4A were performed with WT and ADAP^−/−^ C57BL/6 mice. HE staining of lung tissue was examined on day 26 (representative graph of 5 mice).

B, C PD-1 expression in CD44^hi^CD8^+^ or CD44^hi^CD4^+^ T cells (mean ± SD, *n* ≥ 5 mice per group) or granzyme B expression in CD44^hi^CD8^+^ T cells were examined from the lungs at day 26 (representative data of 3 mice per group).

D, E CTLA-4 levels in lung infiltrating CD44^hi^CD8^+^ T cells and the percentages of CD4^+^Foxp3^+^ T cells in the lungs or MLN were measured at day 26 (mean ± SD, *n* ≥ 5 mice per group).

Data information: Statistical significance was determined with Mann–Whitney *U*-test. Data are representative of three independent experiments.

### SKAP55-deficient mice and SKAP55-deficient CTLs enhance anti-tumor therapy *in vivo*

From a therapeutic perspective, wild-type and SKAP55^−/−^ mice were s.c. injected with E.G7 lymphoma cells to allow tumors to grow for 7 days; OVA_257-264_-pulsed DCs were then injected twice at day 7 and day 14 for therapy (Supplementary Fig S6A). Without immunization of DC vaccine, E.G7 lymphoma cells grew and formed tumors at a similar speed in the PBS-treated wild-type or SKAP55-deficient mice. After the treatment with DCs presenting OVA, wild-type mice showed a modest repression of tumor growth under the skin compared to the PBS-treated controls (Supplementary Fig S6A, red lines). In contrast, SKAP55-deficient mice dramatically blocked tumor growth after DC-induced therapy (Supplementary Fig S6A, blue lines). SKAP55 deficiency also reduced surface PD-1 expression on OVA_257-264_-specific Vα2^high^ CD8^+^ effector cells (Supplementary Fig S6B).

**Figure 6:**
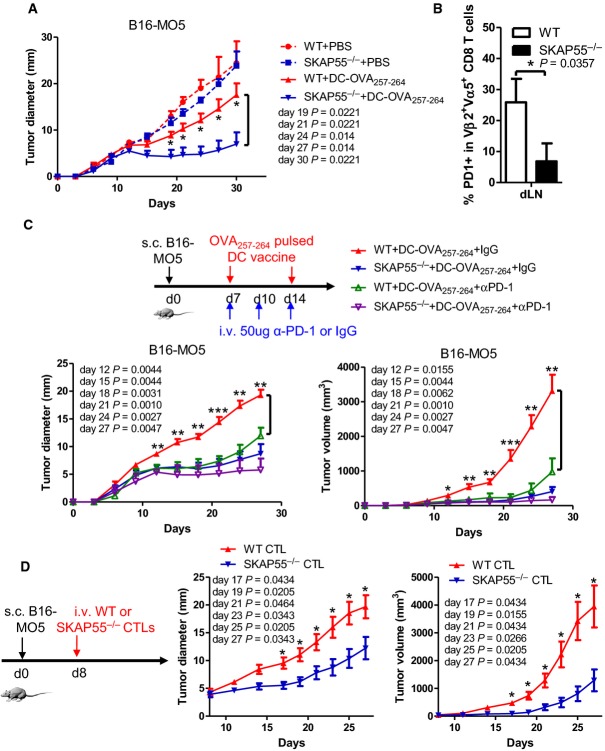
SKAP55-deficient mice and SKAP55-deficient CTLs enhance anti-tumor therapy *in vivo*

We next examined whether SKAP55 deficiency improved DC vaccine-based therapeutic efficacy in melanoma tumor model. The B16F10 tumor cells expressing OVA_257-264_-peptide (i.e., B16-MO5 cells) were s.c. injected into wild-type and SKAP55^−/−^ recipient mice for 7 days; OVA_257-264_-pulsed DCs were then injected twice at day 7 and day 14 for therapy (Supplementary Fig S6A). Indeed, SKAP55-deficient mice significantly repressed melanoma growth and improved DC-induced therapeutic efficacy compared to wild-type controls (Fig6A). SKAP55 deficiency also reduced surface PD-1 expression on OVA_257-264_-specific Vα2^high^/Vβ5^+^ CD8^+^ effector cells (Fig6B). Furthermore, we observed that ADAP-deficient mice and ADAP/SKAP55 DKO mice also significantly repressed melanoma growth after DC vaccination (Supplementary Fig S6C).

A, B WT and SKAP55^−/−^ mice were s.c. injected with MO5 melanoma cells followed by two injection at day 7 and day 14 of OVA_257-264_-pulsed DCs or the PBS control respectively (*n* ≥ 5). The growth of tumors under skin was measured every three days according to tumor diameter (mean ± SEM, *n* ≥ 5 mice per group) (A). Surface expression levels of PD-1 were checked on OVA_257-264_-specific Vα2^high^/Vβ5^+^ CD8^+^ effector cells at day 30 (mean ± SD, *n* ≥ 5 mice) (B).

C WT and SKAP55^−/−^ mice were s.c. injected with MO5 melanoma cells followed by s.c. two injection of OVA_257-264_-pulsed DCs at day 7 and day 14. anti-PD-1 monoantibody or IgG controls were i.v. injected three times at day 7, day 10, and day 14 (50 μg/injection). The diameter and volume of tumors were measured every three days (mean ± SEM, *n* ≥ 5 mice).

D The WT recipient mice were s.c. injected with MO5 melanoma cells followed by injection of 10 nM OVA_257-264_-stimulated WT or SKAP55^−/−^ CTLs at day 8 (*n* ≥ 8). The diameter and volume of tumors were measured every three days (mean ± SEM, *n* ≥ 8 mice).

Data information: Statistical significance was determined with Mann–Whitney *U*-test. Data are representative of two independent experiments.

To examine whether PD-1 blocking improved anti-tumor response, wild-type and SKAP55^−/−^ mice were s.c. injected with B16-MO5 melanoma cells followed by two treatments of OVA_257-264_-pulsed DC vaccine at day 7 and day 14. These mice were also i.v. injected with anti-PD-1 monoantibody at day 7, 10, and 14. Anti-PD-1 monotherapy improved DC-induced anti-tumor therapy in wild-type mice, showing the reduced diameter and volume of tumors (Fig6C, green vs. red lines). Furthermore, anti-PD-1 monotherapy enabled wild-type mice to block tumor growth to similar levels as SKAP55-deficient mice (Fig6C, green vs. purple lines). In contrast, SKAP55-deficient mice showed a minor effect in response to anti-PD-1 monotherapy (Fig6C, purple vs. blue lines).

We next assessed the role of wild-type or SKAP55 KO or ADAP KO CD8^+^ CTLs to suppress tumor growth. To do this, wild-type recipient mice were s.c. injected with B16-MO5 melanoma cells, then OVA_257-264_-stimulated wild-type or SKAP55 KO CD8^+^ CTLs were i.v. injected into these recipient mice at day 8. The diameter and volume of tumors were measured every 3 days, and SKAP55 KO CD8^+^ CTLs (Fig6D) and ADAP KO CD8^+^ CTLs (Supplementary Fig S6D) suppressed tumor growth more effectively than wild-type CD8^+^ CTLs. These results together suggest that except for preventing tumor formation, SKAP55 or ADAP deficiency also improves the efficacy of anti-tumor immunotherapy, which is related to the reduced PD-1 expression.

### The *in vitro* CsA-pretreated CD8^+^ CTLs enhance anti-tumor ability

**Figure 7:**
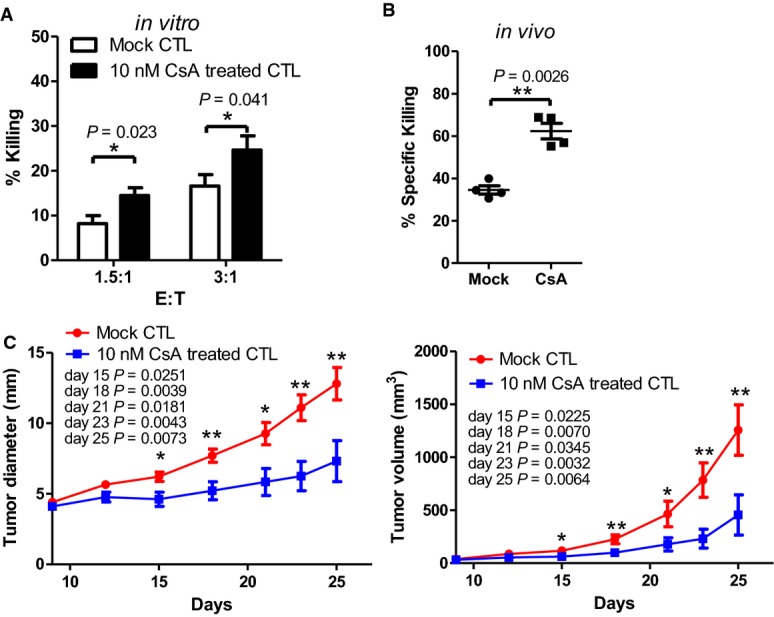
The *in vitro* CsA-pretreated CD8^+^ CTLs enhance anti-tumor ability *in vivo*

Since we showed that the NFATc1 inhibitor CsA treatment significantly decreased the mRNA levels and surface expression of PD-1 in wild-type CD8^+^ CTLs (Fig3D), we tested whether *in vitro* targeting NFATc1 in CD8^+^ CTLs could increase anti-tumor responses. To generate *in vitro* CsA-treated CTLs, WT OT-I splenocytes were cultured with 10 nM OVA_257-264_ and 10 nM CsA for 3 days. Cells were then washed and cultured with RPMI growth medium for 3 days. We found that CsA-pretreated CTLs indeed enhanced their killing ability against OVA_257-264_-pulsed EL4 cells *in vitro* or OVA_257-264_-pulsed splenocytes *in vivo* (Fig7A, B). We then tested whether the *in vitro* CsA-pretreated wild-type CD8^+^ CTLs could suppress melanoma growth more effectively. The wild-type recipient mice were s.c. injected with B16-MO5 melanoma cells, then CsA-treated or untreated OVA_257-264_-specific wild-type CD8^+^ CTLs were i.v. injected into these recipient mice at day 9. Interestingly, tumor diameter and volume were relatively smaller in the recipient mice which received the CsA-pretreated wild-type CD8^+^ CTLs (Fig7C). Together, we propose that the ADAP-SKAP55-NFATc1-PD-1 axis in CD8^+^ CTLs might play crucial role during anti-tumor immunity.

A, B During generation of OVA_257-264_ peptide-specific CTLs, naïve OT-I splenocytes were stimulated with 10 nM OVA_257-264_ with or without CsA (10 nM) for 3 days, then washed, and further cultured in the RPMI growth medium until day 6. The CsA-treated or untreated OVA_257-264_ peptide-specific CTLs were incubated with OVA_257-264_-pulsed EL-4 cells for 4 h to assess the *in vitro* cytotoxicity (A, mean of triplicates ± SD) or i.v. injected into WT mice to measure *in vivo* cytotoxicity (B, mean ± SD, *n* = 4 mice).

C WT mice were s.c. injected with B16-MO5 melanoma cells followed by injection of the CsA-treated or untreated OVA_257-264_ peptide-specific CTLs at day 9 (*n* ≥ 12). The diameter and volume of tumors were measured every 3 days (mean ± SEM, *n* ≥ 12 mice).

Data information: Statistical significance was determined with Mann–Whitney *U*-test. Data are representative of two independent experiments.

## Discussion

Multiple studies have revealed that PD-1 impairs CD8^+^ CTL-induced anti-tumor immune responses (Hamanishi *et al*, 2007; Nomi *et al*, 2007), and anti-chronic viral infections including HIV, HBV, and HCV (Barber *et al*, 2006; Wherry *et al*, 2007). Although blockade of the PD-1/PDL1 pathway in antigen-specific CTLs presents a potential clinical strategy to treat tumor and chronic viral infection (Ha *et al*, 2008; Dotti, 2009; Brahmer *et al*, 2012; Topalian *et al*, 2012), it is not fully understood how PD-1 expression and CD8^+^ CTL cytotoxic efficacy are regulated during immunotherapy. Our study provides the first evidence that SKAP55 or ADAP deficiency greatly enhances CD8^+^ CTL cytotoxicity for tumor prevention or therapy by reducing PD-1 expression (see model in Supplementary Fig S7). In the agreement with previous findings that ADAP stabilizes SKAP55 expression at protein levels, and ADAP KO T cells loss SKAP55 expression (Huang *et al*, 2005; Wang *et al*, 2007), the double knockout mice show similar anti-tumor phenotype as ADAP or SKAP55 knockout mice.

Since ADAP and SKAP55 increase integrin activation in TCR-induced ‘inside-out’ signaling (Griffiths *et al*, 2001; Peterson *et al*, 2001; Wang *et al*, 2003; Jo *et al*, 2005; Kliche *et al*, 2006), and integrin LFA-1 promotes CD8^+^ CTL to form killing synapses with tumor cells to enhance lytic ability (Schmits *et al*, 1996; Franciszkiewicz *et al*, 2013), we unexpectedly observed that loss of ADAP or SKAP55 in CTLs enhanced anti-tumor immunity. Previous data showed that in response to low TCR signaling or suboptimal antigen stimulation, T cells enhance integrin adhesion via the ADAP-SKAP55 complex; and LFA-1-mediated costimulation facilitates T cells to achieve optimal activation. Upon strong antigen stimulation, wild-type cells do not further enhance cell proliferation or adhesion, while ADAP- or SKAP55-deficient T cells enhance proliferation to similar levels as wild-type cells (Mueller *et al*, 2007; Wang *et al*, 2007). The underlying mechanism was not provided by these previous studies. This study shows that SKAP55 and ADAP form an immune complex with Fyn, SHP-2, and PD-1 in antigen-stimulated CTLs. As a kinase, Fyn directly binds and phosphorylates ADAP, SKAP55, and SHP-2, while SHP-2 interacts with PD-1. In agreement with this, another group reported that ADAP forms a complex with SHP-2 and SLP-76 (Pazdrak *et al*, 2008). PD-1 and SHP-2 have been demonstrated to inhibit integrin adhesion (Kwon *et al*, 2005; Saunders *et al*, 2005) and T-cell activation (Chemnitz *et al*, 2004). To reconcile the reduced cytotoxicity with the enhanced integrin activity by SKAP55/ADAP, we propose that in response to strong antigen stimulation, the ADAP-SKAP55 module turns on PD-1 expression, or cooperates with SHP-2, to maintain cell activation and adhesion at an equilibrium or steady state. The ADAP-SKAP55-PD-1 pathway might represent a ‘self-control’ mechanism to control T-cell activation and adhesion precisely.

Our study has demonstrated that the ADAP-SKAP55 module regulates NFATc1 expression/activation to increase PD-1 expression. Given the important role of NFATc1 in T-cell activation, the NFAT inhibitor CsA is used as an immunosuppressive agent in graft-vs-host disease (GVHD) and autoimmune diseases (Macian, 2005). In contrast, we observed that the *in vitro* CsA-pretreated CD8^+^ CTLs reduced PD-1 expression, and injection of the *in vitro* CsA-pretreated CD8^+^ CTLs could enhance the recipient mice against tumor growth. Pretreatment of T cells with CsA before adoptive transfer to tumor patients could be an indication for this drug. Other studies suggest that CsA inhibits tumor growth due to a blockage of cell cycle and the induction of necrosis, supporting the usage of CsA in anti-tumor therapy (Pyrzynska *et al*, 2002; Werneck *et al*, 2012). We propose that the *in vitro* CsA pretreatment of CD8^+^ CTLs and the restricted local injection of low-dose CsA only in solid tumors might limit the immunosuppressive role of CsA. Thus, it is possible to consider a new usage of CsA for anti-tumor therapy by targeting PD-1 expression in CD8^+^ T cells or by targeting tumor itself.

Interestingly, although tumor infiltrating SKAP55 KO or ADAP KO CD8^+^ T cells, as well as CD4^+^ effector/memory T cells, substantially reduced PD-1 expression during anti-tumor response, we did not observe any significant changes in CTLA-4 expression or infiltrating CD4^+^Foxp3^+^ Tregs *in vivo*. It has been shown that PD-1 expression on CD4^+^ effector T cells and the presence of CD4^+^Foxp3^+^ Tregs suppress anti-tumor responses (Ding *et al*, 2012; Mkrtichyan *et al*, 2012). Depletion of Tregs and anti-CTLA-4 mAb treatment are effective and promising anti-tumor immunotherapies (Viguier *et al*, 2004). In addition, although two-thirds of CD8^+^ tumor infiltrating lymphocytes (TIL) express PD-1, one-third to half of CD8^+^ TIL are PD-1/CTLA-4 double-positive that exhibit more severe dysfunction than single-positive (PD-1^+^) TIL (Duraiswamy *et al*, 2013). It is therefore important to investigate which signaling proteins might co-operate with SKAP55 and ADAP to induce PD-1^+^CTLA-4^+^ CD8^+^ TIL in tumors. In addition, it is interesting to investigate whether targeting SKAP55, ADAP, NFATc1 should provide potential benefit for those cancer patients who suffer severe adverse effects of anti-PD1 mAbs (Hansel *et al*, 2010), and whether targeting the ADAP-SKAP55-NFATc1-PD-1 axis might prevent or rescue exhausted CD8^+^ CTLs during chronic viral infection.

## Materials and Methods

### Mice

All mice in this study were on C57BL/6 background. ADAP^−/−^ mice were kindly provided by Dr E. Peterson (University of Minnesota, USA). SKAP55^−/−^ and OT-I TCR transgenic mice were from Dr CE Rudd (University of Cambridge, UK). These mice were bred at 3–5 mice per cage in individual ventilated cages (IVC) under specific pathogen-free conditions at the Animal Care Facility of Shanghai Institute of Biochemistry and Cell Biology, Chinese Academy of Sciences. Mice were segregated by sex, provided with water and rodent chow and maintained under a regular 12-h/12-h light/dark schedule at a constant room temperature (22 ± 2°C). The responsible veterinarian is in charge of the diagnosis, treatment, and control of diseases in the animal facilities. All animal procedures were performed in strict accordance with the institutional guidelines and were approved by the Institutional Animal Care and Use Committee of Shanghai Institute of Biochemistry and Cell Biology (Protocol No. IBCB0057). In all tumor experiments, sample size was estimated according to prior studies. Sex- and age-matched (6–12 weeks old) mice were randomized into the indicated groups. Special attention was taken to determining the humane end points and decided whether the mice should be euthanized in order to avoid further suffering. According to the 3Rs principle, experiments were carefully designed to minimize the use of mice and to obtain maximum amount of data. Animal experiments are reported in accordance with the ARRIVE guidelines (Kilkenny *et al*, 2010).

### Cell lines

The lymphoma EL-4 and OVA-expressing E.G7 cell lines were kind gifts from Dr H Gu (IRCM, Canada). The B16F10 and OVA-expressing B16F10 (i.e. MO5) were melanoma cell lines. EL-4, E.G7, and B16F10 were cultured in Dulbecco's modified Eagle's medium (DMEM) supplemented with 10% (vol/vol) fetal-calf serum and 50 U/ml penicillin–streptomycin. Jurkat cells were cultured in RPMI 1640 supplemented with 10% (vol/vol) fetal-calf serum and 50 U/ml penicillin–streptomycin. All cell lines were not contaminated by mycoplasma, which is confirmed by PCR detection.

### Antibody and reagents

Antibodies against SKAP55 (cat#611236) (BD Pharmingen), ADAP (cat#07-546) (Upstate Biotechnology), NAFTc1 (cat#sc-7294) (Santa Cruz Biotechnology), Fyn (cat#BS2739) (Bioworld Technology), and anti-human CD28 (cat# 348040) (BD Pharmingen) were purchased as indicated. Anti-human CD3 (OKT3) (cat# BE0001-1), anti-mouse CD3 (2C11) (cat#BE0001-1), and anti-mouse CD28 (PV1) (cat#BE0015-5) were from Bio X cell (West Lebanon, NH). Anti-mouse PD-1 PE (cat#12-9981), CTLA4 PE (cat#12-1522), Va2 TCR FITC (cat#11-5812), Vβ5 TCR PerCP-eFluor710 (cat#46-5796), CD44 FITC (cat#11-0441), CD4 PE-Cy7 (cat#25-0041), CD11c FITC (cat#11-0114), CD8a APC (cat#17-0081), CD127 PE (cat#12-1271), F4/80 APC (cat#17-4801), Foxp3 APC (cat#17-5773), Granzyme B FITC (cat#11-8898), and KLRG1 FITC (cat#11-5893) were from eBioscience. Anti-PD-1 (Clone J43) (BE0033-2**) and isotype IgG (BE0091) used in animal experiments were purchased from Bio X cell (West Lebanon, NH). GM-CSF (Peprotech), LPS (lipopolysaccharide, SIGMA), and OVA_257-264_ peptide (SIINFEKL, AnaSpec) were ordered.

### Generation of CD8^+^ CTLs and cytotoxicity assay *in vitro* or *in vivo*

To generate OVA_257-264_ peptide-specific CD8^+^ CTLs, naïve CD8^+^ T cells were isolated from wild-type, ADAP^−/−^, SKAP55^−/−^ OT-I mice with BD IMag™ CD8^+^ Magnetic Particles, followed by co-culture with 20 U/ml human recombinant IL-2 and 10 nM OVA_257-264_ peptide-pulsed wild-type splenocytes at the ratio of 1:5 for 3 days. To generate the CsA-treated CTLs, WT OT-I splenocytes were cultured with 10 nM OVA_257-264_ and 10 nM CsA for 3 days. Cells were then washed and cultured with RPMI growth medium for 3 days. These *in vitro* activated OT-I CTL were harvested at day 6 and used for cytotoxicity assay. For an *in vivo* killing assay, CD8^+^ CTLs (3 × 10^6^) were i.v. injected into the recipient C57BL/6 mice. After 4 hours, CFSE^lo^ (1 μM)-labeled splenocytes were pulsed with 10 nM OVA_257-264_ peptide and mixed at 1:1 ratio with unpulsed CFSE^hi^ (10 μM)-labeled splenocytes (5 × 10^6^), and i.v. injected into the recipient mice for 6 h. Splenocytes from recipient mice were analyzed by flow cytometry, and cytotoxicity was calculated as [1− (% CFSE^lo^)/(% CFSE^hi^)] × 100%. For *in vitro* cytotoxicity assay, EL-4 lymphoma cells (1 × 10^4^) were pulsed with 10 nM OVA_257-264_ peptide for 1 h and cocultured with CD8^+^ CTLs at different cell ratios in phenol red-free medium for 4 h. Lactate dehydrogenase (LDH) in the supernatants was quantified by CytoTox 96 Non-Radioactive Cytotoxicity Assay (Promega).

### DC vaccination for prevention of tumor

Bone marrow cells were cultured with GM-CSF (10 ng/ml) for 7 days, then stimulated with 100 ng/ml LPS for 24 h to generate mature BM-derived DCs. DCs were pulsed with 10 nM OVA_257-264_ peptide for 2 h or with B16F10 tumor cells lysates at a ratio of three tumor cells to one DC overnight, followed by mitomycin C treatment (20 μg/ml) for 2 h. After extensive wash, these DCs were resuspended in PBS, and injected s.c. on day 0, and boosted on day 7 at 3 sites in low flanks (1 × 10^5^/100 μl) of the recipient mice. 7 days after the second immunization, mice were challenged with E.G7 (5 × 10^5^/100 μl, s.c.) or B16F10 (2 × 10^5^/100 μl, i.v.), and tumor size was measured every three days by an investigator blinded to the information of the mice.

### DC-based anti-tumor therapy

Mice were s.c. injected with E.G7 lymphoma (1 × 10^6^/100 μl PBS) or MO5 melanoma cells (2 × 10^5^/100 μl). On day 7 and 14, 10 nM OVA_257-264_-pulsed DCs were s.c. injected into these mice at 3 sites in low flanks (1 × 10^5^/100 μl) of the recipient mice and the size of tumors was recorded every three days by an investigator blinded to the information of the mice. Alternatively, these mice were i.v. treated with 50 μg anti-PD-1 monoantibody (clone J43) or the control IgG antibodies at day 7, 10, and 14 to repress tumor growth.

### Injection of CD8^+^ CTLs for anti-tumor therapy

Wild-type, SKAP55-deficient, ADAP-deficient or CsA-treated CD8^+^ OT-I CTLs were generated with OVA_257-264_ peptide stimulation as the methods described above. Wild-type C57BL6 mice were used as recipient mice and s.c. injected with MO5 melanoma cells (2 × 10^5^/100 μl). At day 8 to 10 post-injection of MO5 cells (i.e. the tumors were palpable), 1 × 10^6^ OVA_257-264_ peptide-specific wild-type or SKAP55-deficient, ADAP-deficient or CsA-treated CD8^+^ OT-I CTLs were i.v. injected into the recipient mice, and the tumor growth was recorded every three days by an investigator blinded to the information of the mice.

### Flow cytometry and immunofluorescence

For intracellular staining, wild-type, SKAP55^−/−^, or ADAP^−/−^ CD8^+^ CTLs were fixed with 2% paraformaldehyde (PFA), permeabilized with 0.1% Triton X-100, then labeled with the indicated antibodies in FACS buffer (PBS/1%FCS/0.02%NaN_3_). For surface staining, cells were labeled with the indicated Abs in FACS buffer and analyzed using FACS Calibur or C6 (BD Bioscience). To stain the killing synapses, EL-4 cells were pulsed with 10 nM OVA_257-264_ peptide for 1 h (4 × 10^6^ cells/ml), then mixed with CD8^+^ CTLs at 1:1 ratio for 30 min. Conjugates were fixed with PFA and permeabilized with 0.1% Triton X-100, followed by staining with the indicated antibodies. Nuclei were stained with Hoechst, and images were taken under Olympus IX81 microscopy.

### Western bolt and immunoprecipitation

Wild-type, SKAP55^−/−^, or ADAP^−/−^ CD8^+^ CTLs were left unstimulated or stimulated with 10 nM OVA_257-264_-pulsed EL-4 for 4 h. Cell pellets were lysed for immunoblotting with anti-mouse NFATc1 and β-actin. For immunoprecipitation, CD8^+^ CTLs were stimulated with hydrogen peroxide and sodium orthovanadate. Cell lysates were incubated with various antibodies and protein G-coupled sepharose beads (Sigma) respectively, followed by immunoblotting with antibodies against SKAP55, ADAP, and Fyn.

### Real-time PCR and EMSA

Total RNA was extracted from cells with Trizol reagent (Sigma). cDNA was prepared with a reverse-transcriptase M-MLV kit (Takara). Real-time PCR was performed on a CFX-96 machine (Bio-Rad) with SYBR Green Master Mix (DBI Bioscience). The housekeeping gene 18S rRNA was used for normalization. Primer sequences are listed in Supplementary Table S1. The EMSA was performed as previously described (Oestreich *et al*, 2008). For EMSA, nuclear extracts were prepared from wild-type, SKAP55^−/−^, or ADAP^−/−^ CTLs. Biotin-labeled double-stranded NFATc1 binding DNA probe N1 (5′-GCTTGGTGGGGAAGGAAACATTACTTTGAA-3′) was incubated with 5 μg nuclear extract for 30 min in 20 μl reaction buffer containing 100 ng double-stranded poly(dI:dC) and 50 mM DTT. For competition experiments, nuclear extracts were pre-incubated for 20 min with a 100-fold molar excess of the unlabeled competitor probe (Comp. N1) or the mutant probe that contains the same sequence except for carrying a mutated NFAT-binding site (Comp. mutN1). The protein–DNA complex was separated by electrophoresis (6% nondenaturing TBE gels) and detected by a Chemiluminescent EMSA kit (Beyotime).

### Luciferase report assay

The pGL3-NFAT Luciferase reporter plasmid contains 3× NFAT-binding sequence in the IL-2 promoter. Jurkat cells were transfected with the NFAT-luciferase reporter and plasmids expressing GFP, pEGFP-SKAP55, pEGFP-ADAP, or pEGFP-SKAP55ΔSH3. Cells were then stimulated with 2 μg/ml anti-CD3 and 4 μg/ml anti-CD28 antibodies for 6–8 h, followed by measurement of luciferase activity using Dual-Glo luciferase system (Gibco).

### Statistical analysis

All experiments were performed at least twice. For the animal experiments, a nonparametric distribution was assumed, and statistical significance was assessed with Mann–Whitney *U*-test. For the other experiments, unpaired two-tailed Student's *t*-test was used to compare two groups. Statistical significance was marked as ‘*’ when *P* < 0.05, ‘**’ when *P* < 0.01, or ‘***’ when *P* < 0.001. *P* values <0.05 were considered statistically significant.

## For more information

Author's website: http://www.sibcb.ac.cn/ePI.asp?id=132

## Supporting Information

Supplementary Table S1 Supplementary Figures S1–S7

Review Process File
