# Supplementary material for: ADAP and SKAP55 deficiency suppresses PD-1 expression in CD8+ cytotoxic T lymphocytes for enhanced anti-tumor immunotherapy
Source: EMBO Mol Med. 2015 Apr 7;7(6):754–69. doi: 10.15252/emmm.201404578 (PMC4459816; doi:10.15252/emmm.201404578)
Supplement: Supplementary file 1 [file emmm0007-0754-sd1.pdf]

Supplementary information

ADAP and SKAP55 deficiency suppresses PD-1 expression on CD8^{+} cytotoxic T lymphocytes for enhanced anti-tumor immunotherapy

Chunyang Li[1], Weiyun Li[1], Jun Xiao[1,2], Shaozhuo Jiao[1], Fei Teng[1], Shengjie Xue[1], Chi Zhang[1], Chun Sheng[2], Qibin Leng[3], Christopher E. Rudd[4], Bin Wei[5#], Hongyan Wang[1#]

Table of content

Supplementary Table S1: RT-PCR Primers
Supplementary Figure S1
Supplementary Figure S2
Supplementary Figure S3
Supplementary Figure S4
Supplementary Figure S5
Supplementary Figure S6
Supplementary Figure Legends

Supplementary Table S1: RT-PCR Primers

	Sequences
Blimp1 Forward	5’-GACGGGGGTACTTCTGTTCA
Blimp1 Reverse	5’-GGCATTCTTGGGAACTGTGT
c-Fos Forward	5’-GGGACAGCCTTTCCTACTAC
c-Fos Reverse	5’-GGGATAAAGTTGGCACTAGAG
CD25 Forward	5‘-AACCATAGTACCCAGTTGTCGG
CD25 Reverse	5’-TCCTAAGCAACGCATATAGACCA
CD69 Forward	5'-CCCTTGGGCTGTGTTAATAGTG
CD69 Reverse	5'-AACTTCTCGTACAAGCCTGGG
GranzymeB Forward	5’-CCACTCTCGACCCTACATGG
GranzymeB Reverse	5’-GGCCCCCAAAGTGACATTTATT
IL-2 Forward	5’-GGAGCAGCTGTTGATGGACCTAC
IL-2 Reverse	5’-AATCCAGAACATGCCGCAGAG
IFN-γ Forward	5'-ATGAACGCTACACACTGCATC
IFN-γ Reverse	5'-CCATCCTTTTGCCAGTTCCTC
NFATc1 Forward	5’-GACCCGGAGTTCGACTTCG
NFATc1 Reverse	5’-TGACACTAGGGGACACATAACTG
PD-1 Forward	5’-GCACCCCAAGGCAAAAATCG
PD-1 Reverse	5’-CAATACAGGGATACCCACTAGGG
Peforin Forward	5'-AGCACAAGTTCGTGCCAGG
Peforin Reverse	5'-GCGTCTCTCATTAGGGAGTTTTT
18s Forward	5’-AGTTCCAGCACATTTTGCGAG
18s Reverse	5’-TCATCCTCCGTGAGTTCTCCA

Figure S1:

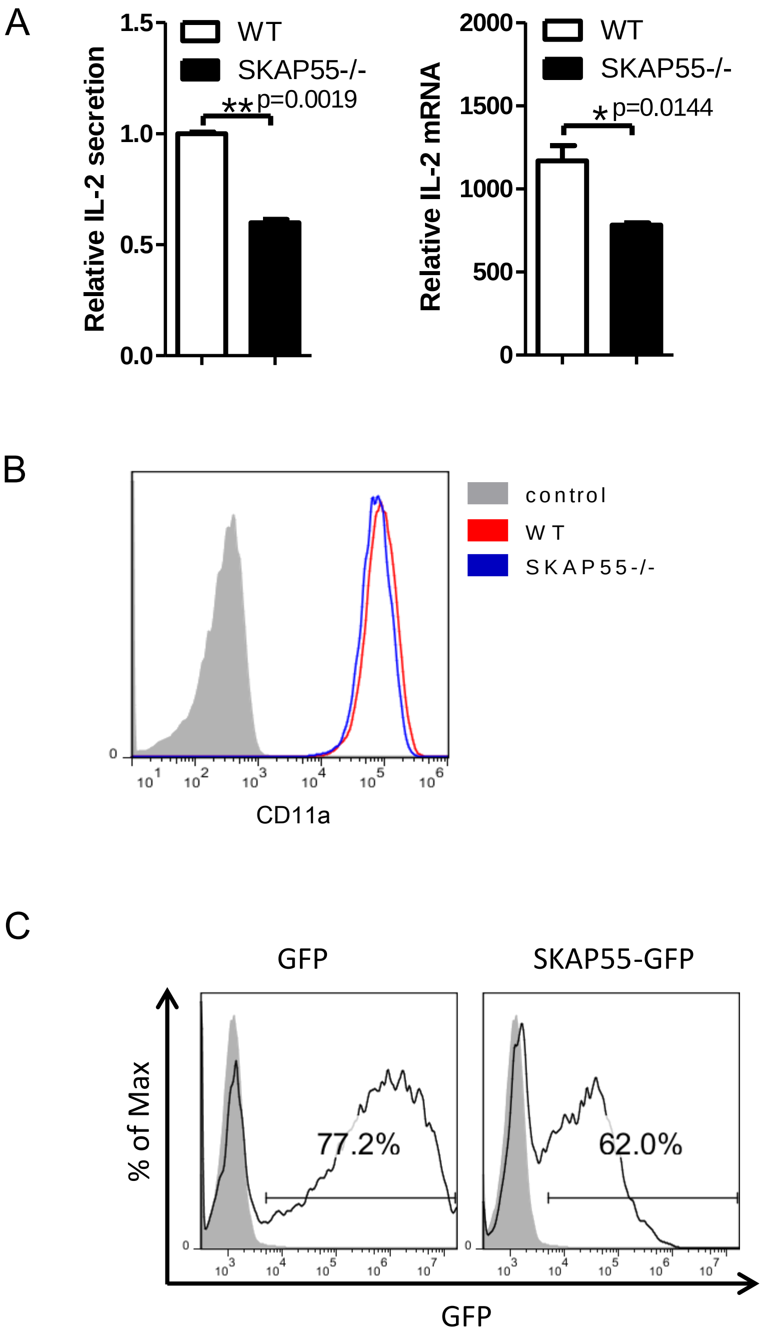
A
Relative IL-2 secretion
1.5
1.0
0.5
0.0
WT
SKAP55-/-
** p=0.0019
Relative IL-2 mRNA
2000
1500
1000
500
0
WT
SKAP55-/-
* p=0.0144
B
control
WT
SKAP55-/-
CD11a
C
GFP
SKAP55-GFP
% of Max
77.2%
62.0%
GFP

Figure S2:

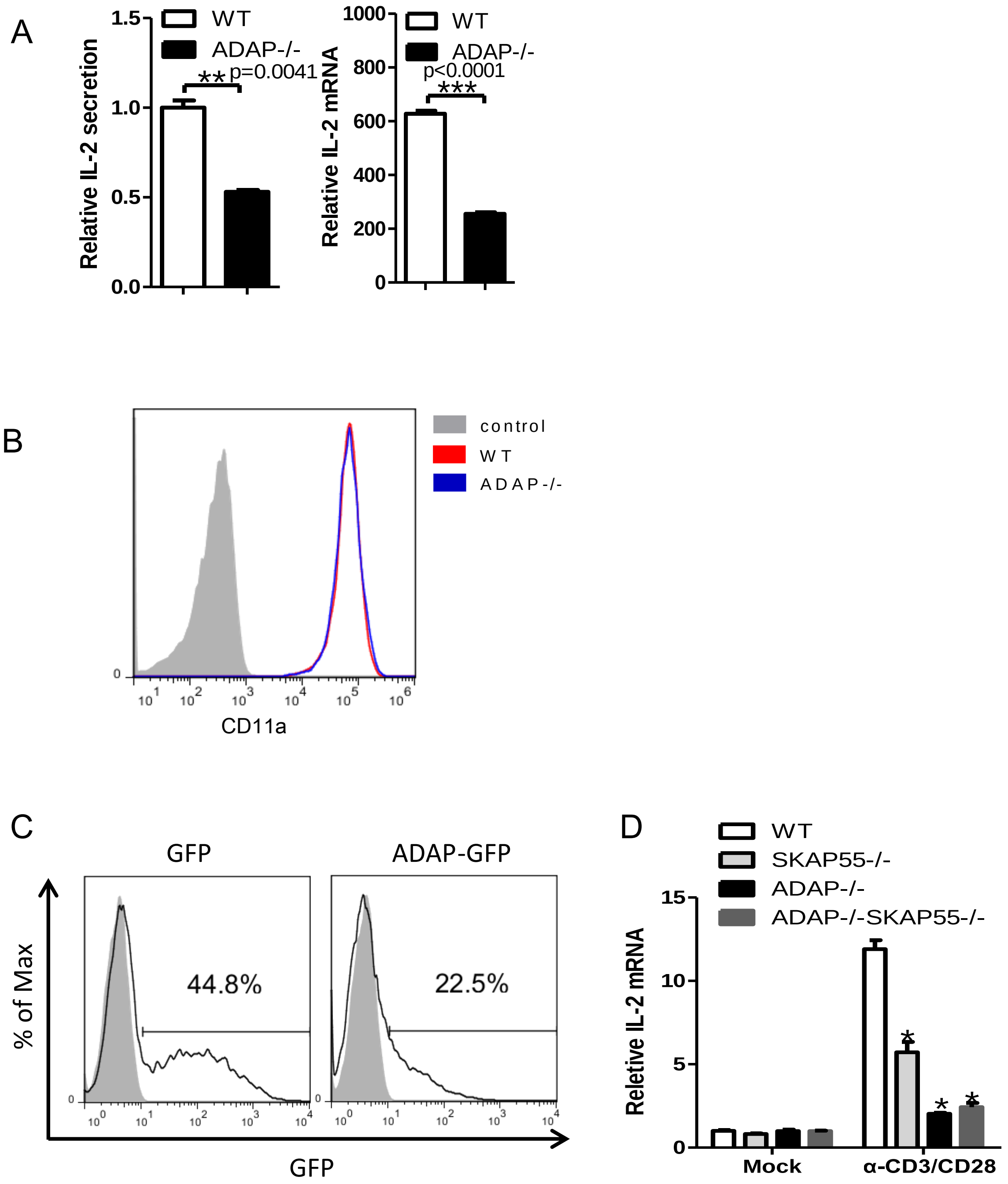
A
Relative IL-2 secretion
1.5
1.0
0.5
0.0
WT
ADAP-/-
**
p=0.0041
Relative IL-2 mRNA
1000
800
600
400
200
0
p<0.0001
***
B
control
WT
ADAP-/-
CD11a
C
GFP
ADAP-GFP
% of Max
44.8%
22.5%
GFP
D
WT
SKAP55-/-
ADAP-/-
ADAP-/-SKAP55-/-
Reletive IL-2 mRNA
15
10
5
0
Mock
α-CD3/CD28

Figure S3:

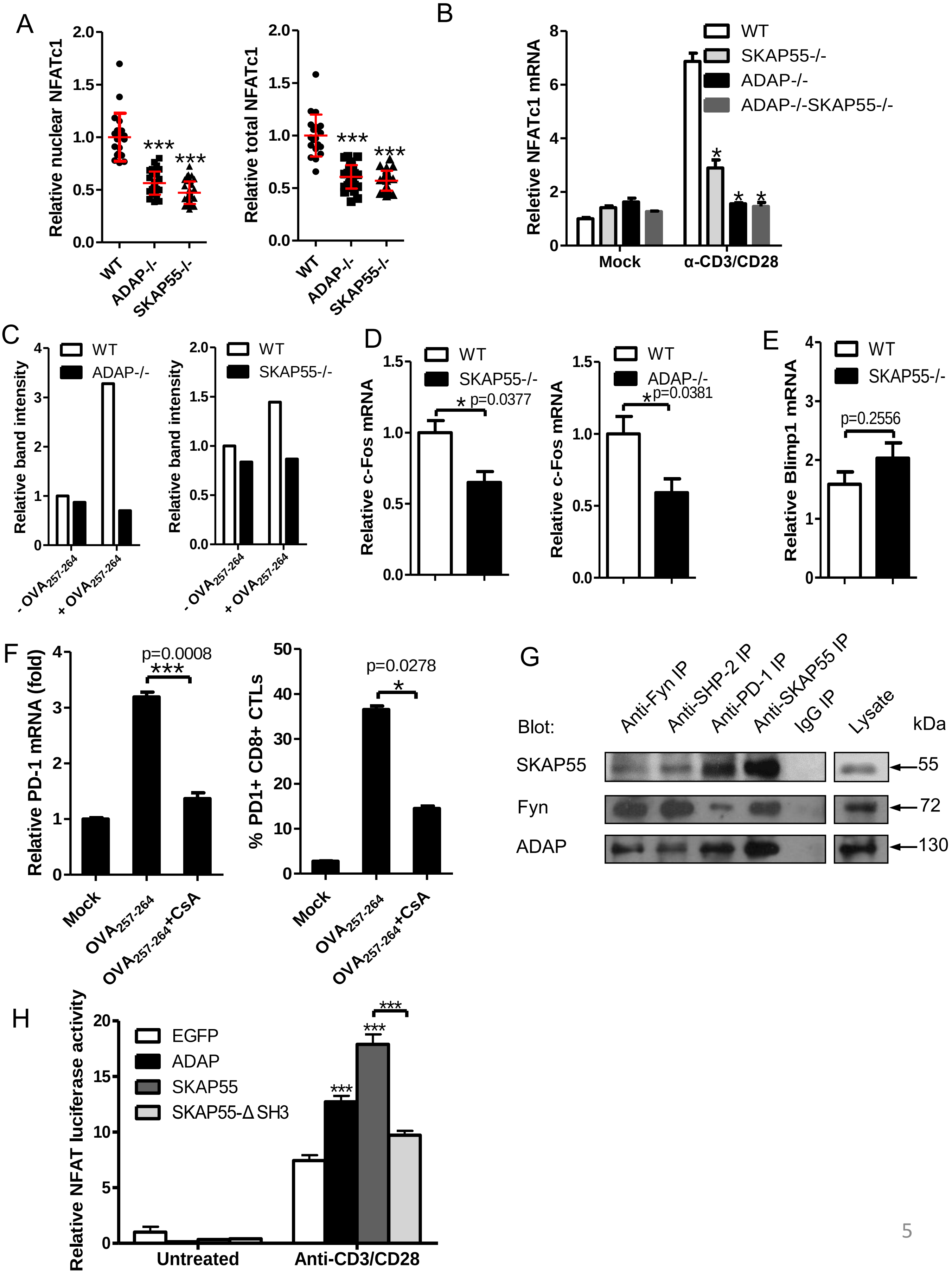
A
Relative nuclear NFATc1
Relative total NFATc1
WT
ADAP-/-
SKAP55-/-
***
B
Relative NFATc1 mRNA
WT
SKAP55-/-
ADAP-/-
ADAP-/-SKAP55-/-
Mock
α-CD3/CD28
C
Relative band intensity
WT
ADAP-/-
SKAP55-/-
- OVA257-264
+ OVA257-264
D
Relative c-Fos mRNA
WT
SKAP55-/-
ADAP-/-
p=0.0377
p=0.0381
E
Relative Blimp1 mRNA
WT
SKAP55-/-
p=0.2556
F
Relative PD-1 mRNA (fold)
p=0.0008
% PD1+ CD8+ CTLs
p=0.0278
Mock
OVA257-264
OVA257-264+CsA
G
Blot:
Anti-Fyn IP
Anti-SHP-2 IP
Anti-PD-1 IP
Anti-SKAP55 IP
IgG IP
Lysate
kDa
SKAP55
Fyn
ADAP
55
72
130
H
Relative NFAT luciferase activity
EGFP
ADAP
SKAP55
SKAP55-Δ SH3
Untreated
Anti-CD3/CD28

Figure S4:

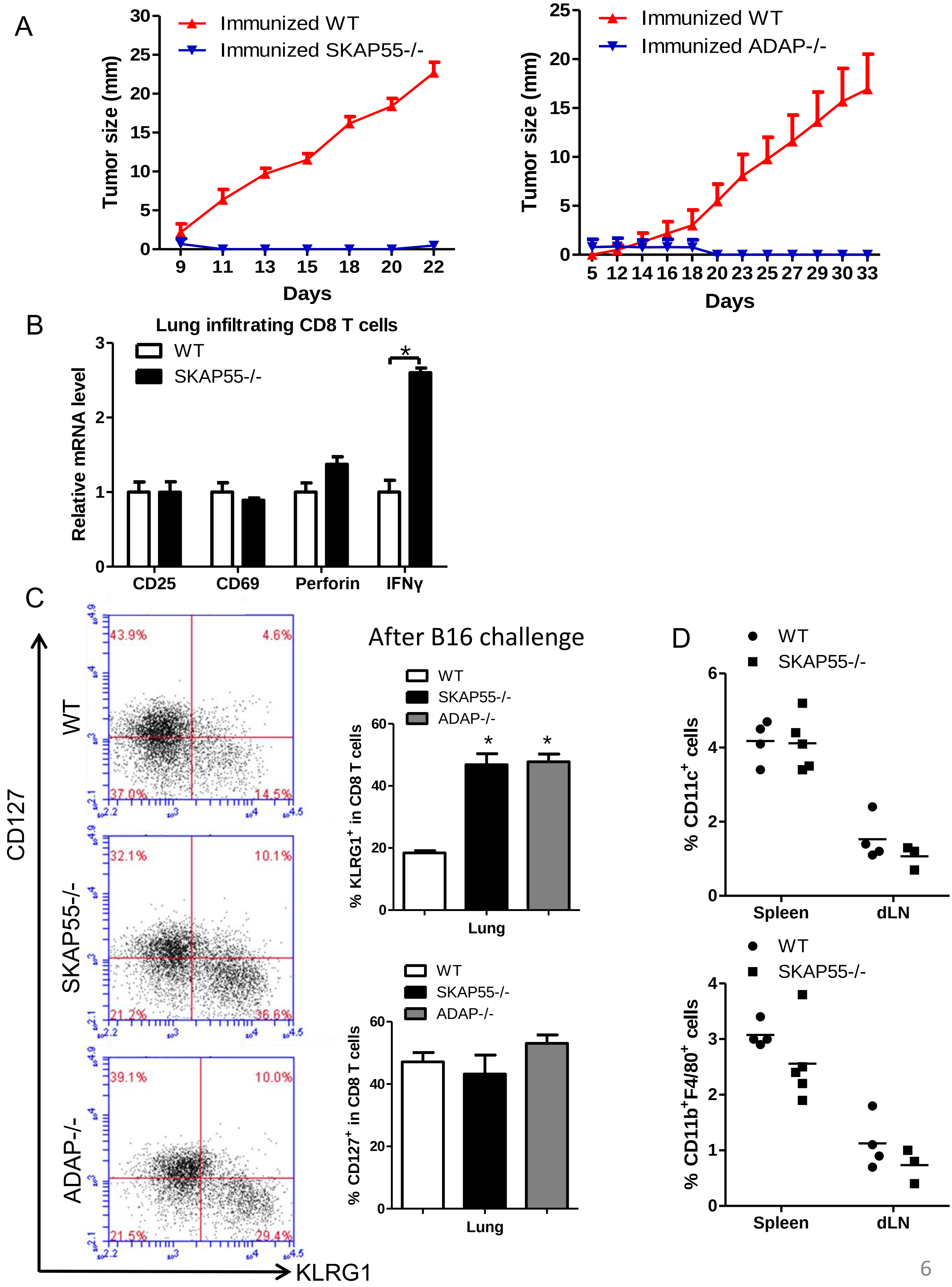
A
Immunized WT
Immunized SKAP55-/-
Tumor size (mm)
Days
Immunized WT
Immunized ADAP-/-
Tumor size (mm)
Days
B
Lung infiltrating CD8 T cells
WT
SKAP55-/-
Relative mRNA level
CD25
CD69
Perforin
IFNγ
C
CD127
KLRG1
WT
SKAP55-/-
ADAP-/-
43.9%
4.6%
37.0%
14.5%
32.1%
10.1%
21.2%
36.6%
39.1%
10.0%
21.5%
29.4%
After B16 challenge
WT
SKAP55-/-
ADAP-/-
% KLRG1+ in CD8 T cells
Lung
% CD127+ in CD8 T cells
Lung
D
WT
SKAP55-/-
% CD11c+ cells
Spleen
dLN
% CD11b+F4/80+ cells
Spleen
dLN

Figure S5:

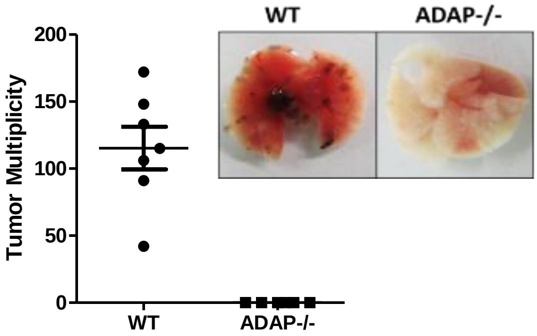

Figure S6:

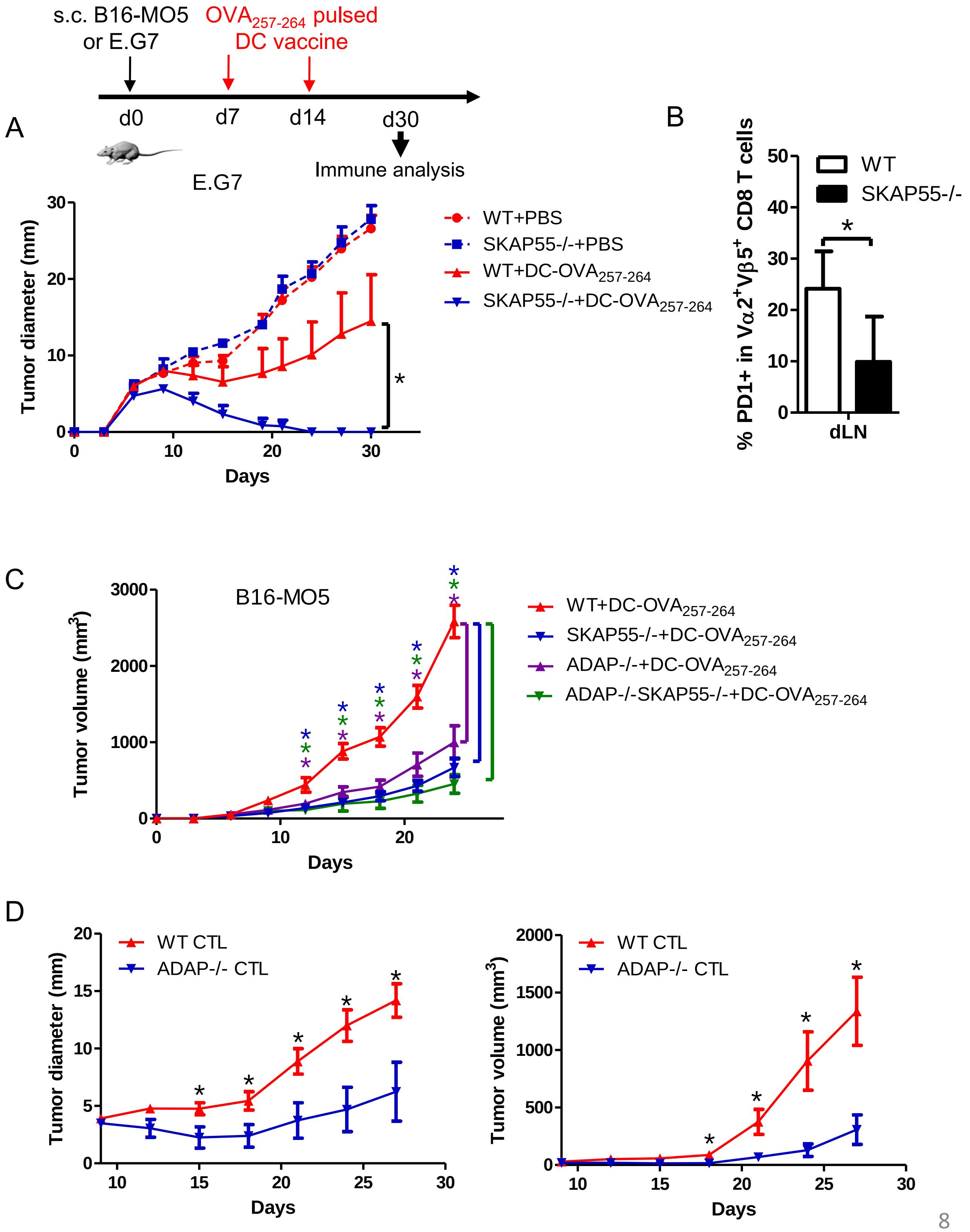

s.c. B16-MO5 or E.G7
OVA257-264 pulsed DC vaccine
d0
d7
d14
d30
Immune analysis
A
E.G7
Tumor diameter (mm)
Days
WT+PBS
SKAP55-/-+PBS
WT+DC-OVA257-264
SKAP55-/-+DC-OVA257-264
B
% PD1+ in Vα2+Vβ5+ CD8 T cells
WT
SKAP55-/-
dLN
C
B16-MO5
Tumor volume (mm3)
Days
WT+DC-OVA257-264
SKAP55-/-+DC-OVA257-264
ADAP-/-+DC-OVA257-264
ADAP-/-SKAP55-/-+DC-OVA257-264
D
Tumor diameter (mm)
Tumor volume (mm3)
Days
WT CTL
ADAP-/- CTL

Figure S7:

Model: The ADAP-SKAP55-PD-1 pathway reduces CD8+ CTL cytotoxicity

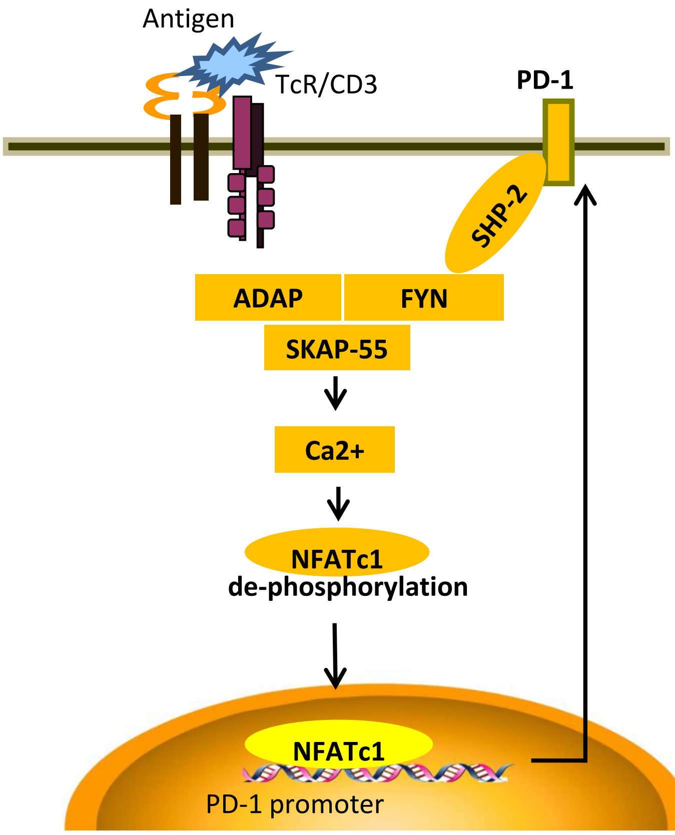

Supplemental Figure Legends

Table S1: The primers for RT-PCR were listed.

Figure S1:

(A) WT and SKAP55$^{-/-}$ OT-I CD8^{+} T cells were stimulated with 10nM OVA$_{257-264}$ peptide for 24 hrs to detect IL-2 expression by ELISA and RT-PCR (mean of triplicates ± SD).

(B) Surface expression CD11a in WT and SKAP55$^{-/-}$ OT-I CD8^{+} CTLs.

(C) CD8^{+} CTLs were transfected with plasmids expressing SKAP55-GFP or EGFP. The expression levels of SKAP55-GFP or GFP were assessed 16hr after transfection.

Statistical significance was determined with unpaired two-tailed Student's t-test. Graphs are representative of three independent experiments.

Figure S2:

(A) WT and ADAP$^{-/-}$ OT-I CD8^{+} T cells were stimulated with 10nM OVA$_{257-264}$ peptide for 24 hrs to measure IL-2 expression (mean of triplicates ± SD).

(B) Surface expression of CD11a in WT and ADAP $^{-/-}$ OT-I CD8^{+} CTLs.

(C) CD8^{+} CTLs were transfected with plasmids expressing ADAP-GFP or EGFP. The expression levels of ADAP-GFP or GFP were assessed 16hr after transfection.

(D) Naïve CD8^{+} T cells from WT, SKAP55$^{-/-}$, ADAP$^{-/-}$ and SKAP55$^{-/-}$ADAP$^{-/-}$ mice was stimulated with plated bound anti-CD3/CD28 for 12hr to measure IL-2 mRNA levels (mean of triplicates ± SD).

Statistical significance was determined with unpaired two-tailed Student's t-test. Graphs are representative of at least three independent experiments.

Figure S3:

(A, C-E) WT, ADAP$^{-/-}$ or SKAP55$^{-/-}$ OT-I CD8^{+} CTLs were stimulated with OVA$_{257-264}$-pulsed or unpulsed EL4 cells. The levels of nuclear and total NFATc1 were examined by immunostaining (A); In an EMSA assay, nuclear extracts from WT and SKAP55$^{-/-}$ or ADAP$^{-/-}$ CTLs were incubated with the DNA probes containing NFAT-binding sites (C); (D, E) The mRNA levels of c-Fos or Blimp-1 were measured 4 hrs

post stimulation (mean of triplicates ± SD).

(B) Naïve CD8$^+$ T cells from WT, SKAP55$^{-/-}$, ADAP$^{-/-}$ and SKAP55$^{-/-}$ADAP$^{-/-}$ mice was stimulated with plated bound anti-CD3/CD28 for 12hr to measure NFATc1 mRNA levels (mean of triplicates ± SD).

(F) Wild type OT-I CTLs were pre-treated with or without CsA (5uM) for 1hr, stimulated by 10nM OVA$_{257-264}$-pulsed EL-4 for 4hrs to assess surface expression and the mRNA levels of PD-1 (mean of triplicates ± SD).

(G) Cell lysates from OT-I CD8$^+$ CTLs were prepared and immunoprecipitated with various antibodies, followed by immunoblotting with antibodies against Fyn, SKAP55 or ADAP.

(H) GFP, ADAP, SKAP55 or SKAP55-ΔSH3 was overexpressed with pGL3-NFAT Luciferase reporter plasmid into Jurkat cells. Cells were stimulated with anti-CD3 and anti-CD28 for 6 hrs, followed by measuring luciferase readings (mean of triplicates ± SD).

Statistical significance was determined with unpaired two-tailed Student's t-test. Graphs are representative of three independent experiments.

Figure S4:

(A) After immunization with OVA$_{257-264}$-pulsed DCs two times, WT or SKAP55$^{-/-}$ or ADAP$^{-/-}$ mice were s.c. challenged with E.G7 cells. Tumor size was recorded (mean ± SEM, n=9 mice per group).

(B-D) WT, SKAP55$^{-/-}$ or ADAP$^{-/-}$ mice were s.c. immunized on day -14 and day -7 by DCs that were pre-pulsed with B16F10 tumor lysates. On day 0, the immunized mice were i.v. inoculated with B16F10 cells (n>=3 mice). The mRNA levels of CD25, CD69, Perforin and IFN-γ in lung infiltrating CD8$^+$ T cells were examined at day 26 (mean of triplicates ± SD). 26 days after B16F10 challenge, we examined the percentage of KLRG1$^+$CD8$^+$ and CD127$^+$CD8$^+$ T cells from lungs (mean ± SEM, n>=3 mice per group) (C) and the percentages of CD11c$^+$ and CD11b$^+$ cells from spleens and dLNs (D).

Statistical significance was determined with Mann–Whitney U test. Data are

representative of three independent experiments.

Figure S5:

WT and ADAP$^{-/-}$ mice were s.c. immunized on day -14 and day -7 by DCs that were pre-pulsed with B16F10 tumor lysates. On day 0, the immunized mice were i.v. inoculated with B16F10 cells. Numbers of lung tumors were examined at day 26. Data are representative of two independent experiments.

Figure S6:

(A, B) WT and SKAP55$^{-/-}$ mice were s.c. injected with EG.7 cells followed by two injections at day 7 and day 14 of OVA$_{257-264}$-pulsed DCs or the PBS control respectively (n>=5). Tumor diameter was measured every three days (mean ± SEM, n>=5 mice per group) (A); Surface expression levels of PD-1 were checked on OVA$_{257-264}$-specific Vα2^{high}/Vβ5^{+} CD8^{+} effector cells at day 30 (mean ± SD, n>=5 mice per group) (B). Data are representative of two independent experiments.

(C) WT, SKAP55$^{-/-}$, ADAP$^{-/-}$ and SKAP55$^{-/-}$ADAP$^{-/-}$ mice were s.c. injected with MO5 melanoma cells followed by two injections of OVA$_{257-264}$-pulsed DCs at day 7 and day 14 respectively (n>=5). The growth of tumors under skin was measured every three days according to tumor diameter (mean ± SEM, n>=5 mice).

(D) The WT recipient mice were s.c. injected with MO5 melanoma cells followed by injection of 10nM OVA$_{257-264}$ stimulated WT or ADAP$^{-/-}$ CTLs at day 9 (n>=8). The diameter and volume of tumors were measured every three days (mean ± SEM, n>=8 mice)

Statistical significance was determined with Mann–Whitney U test. Data are representative of two independent experiments.

Figure S7: Model: The ADAP-SKAP55-NFATc1-PD-1 pathway reduces CD8^{+} CTL cytotoxicity.
