# Supplementary material for: ADAP and SKAP55 deficiency suppresses PD-1 expression in CD8+ cytotoxic T lymphocytes for enhanced anti-tumor immunotherapy
Source: EMBO Mol Med. 2015 Apr 7;7(6):754–69. doi: 10.15252/emmm.201404578 (PMC4459816; doi:10.15252/emmm.201404578)
Supplement: Supplementary file 2 [file emmm0007-0754-sd2.pdf]

EMBO
Molecular Medicine

ADAP and SKAP55 deficiency suppresses PD-1 expression in CD8+ cytotoxic T lymphocytes for enhanced anti-tumor immunotherapy

Chunyang Li, Weiyun Li, Jun Xiao, Shaozhuo Jiao, Fei Teng, Shengjie Xue, Chi Zhang, Chun Sheng, Qibin Leng, Christopher E. Rudd, Bin Wei, Hongyan Wang

Corresponding author: Hongyan Wang, Shanghai Institutes for Biological Sciences

Review timeline:

Submission date:	27 August 2014
Editorial Decision:	24 September 2015
Revision received:	27 January 2015
Editorial Decision:	13 February 2015
Accepted:	04 March 2015

Transaction Report:

(Note: With the exception of the correction of typographical or spelling errors that could be a source of ambiguity, letters and reports are not edited. The original formatting of letters and referee reports may not be reflected in this compilation.)

Editor: Roberto Buccione

1st Editorial Decision 24 September 2015

Thank you for the submission of your manuscript to EMBO Molecular Medicine. We have now heard back from the three Reviewers whom we asked to evaluate your manuscript.

You will see that the Reviewers are clearly supportive of you work, although they do express a number of concerns that prevent us from considering publication at this time. I will not dwell into much detail, as the evaluations are self-explanatory. I would like, however, to specifically address a few main points.

Reviewer 1, in addition to mentioning a number of technical and lexical issues that require your action, mentions a few specific issues. S/he would like to see the data on the extent of over-expression of SKAP55 and on the double knock-outs. Finally, Reviewer 1 also suggests that the figure illustrating the data on tumour protection be made a main one and mentions that survival, as opposed to the numbers of metastases is not a good indicator.

Reviewer 2 similarly to Reviewer 1, suggests careful revision for English usage. S/he also mentions two specific and serious issues for your action. First, s/he suggests that the statistical analysis of your results should be redone with a better-suited approach. In this respect I would suggest seeking the advice of a statistician if necessary. The other concern raised by Reviewer 2 is an ethical one with respect to animal experimentation; in fact, assessing survival as an endpoint instead of focusing on lung cancer multiplicity, contrasts with current ethical guidelines, which as you know recommend choosing endpoints that minimise animal distress and pain. We agree with this Reviewer that the survival curves should not be shown nor these experiments reported. In this

respect the request by Reviewer 1 to provide better lung tumour data is especially fitting. I must also ask you to provide more details in the Materials and Methods section identifying the institutional and/or licensing committee approving the experiments, also including any relevant details (like how many animals were used, of which gender, at what age, which strains, if genetically modified, on which background, housing details, etc). Please note that we encourage authors to follow the ARRIVE guidelines for reporting studies involving animals. Please see the EQUATOR website for details: http://www.equator-network.org/reporting-guidelines/improving-bioscience-research-reporting-the-arrive-guidelines-for-reporting-animal-research/. Connected to this and the above-mentioned statistics issue, we are now requesting all Authors to complete an Author checklist (see below). Finally, do consider that from time to time, where deemed necessary, we consult with external advisors on statistics and ethical issues pertaining to animal and/or human experimentation.

Reviewer 3 is also positive and raises one interesting point concerning cyclosporine A (CSA), which was also mentioned by Reviewer 2. Specifically, s/he would like to see further experimentation to establish significance and would like you, ideally, to compare the effect of CSA with ADAP-SAKP55 deficiency. I agree with him/her that this would significantly increase the relevance and impact of your manuscript and therefore encourage you to develop your study in this sense as far as realistically possible.

Considered all the above, while publication of the paper cannot be considered at this stage, we would be pleased to consider a revised submission, with the understanding that the Reviewers' concerns must be addressed as outlined above, with additional experimental data where appropriate and that acceptance of the manuscript will entail a second round of review.

Please note that it is EMBO Molecular Medicine policy to allow a single round of revision only and that, therefore, acceptance or rejection of the manuscript will depend on the completeness of your responses included in the next, final version of the manuscript.

As you know, EMBO Molecular Medicine has a "scooping protection" policy, whereby similar findings that are published by others during review or revision are not a criterion for rejection. However, I do ask you to get in touch with us after three months if you have not completed your revision, to update us on the status. Please also contact us as soon as possible if similar work is published elsewhere.

Last, but not least important and as mentioned above, please note that EMBO Molecular Medicine now requires a complete author checklist (http://embomolmed.embopress.org/authorguide#editorial3) to be submitted with all revised manuscripts (also attached to this letter).

I look forward to seeing a revised form of your manuscript as soon as possible.

***** Reviewer's comments *****

Referee #1 (Comments on Novelty/Model System):

There is no doubt that the area of PD-1 modulation of anti-tumor immunity is currently of intense and highly competitive interest with respect to translational immunology impacts. This paper offers some new insights and may possibly offer some new therapeutic targets. The reliance on the slightly 'easy win' of the B16 melanoma model possibly diminishes slightly ones enthusiasm for adequacy of the model system

Referee #1 (Remarks):

In this manuscript by Li and colleagues a number of substantive new insights are offered about the role of the SKAP-55/ADAP pathway in modulation of PD-1 expression and thus, the potential for modulatory effects on tumor killing. The points are novel and in many respects, well evidenced by the data.

I have a number of specific comments:

The Abstract could be considerably sharpened. The wording was sometimes ambiguous. For example, in line 5 where it is stated that "KO mice profoundly prevented tumor formation", this needs to be rephrased as something like "knockout of SKAP 55 or ADAP in mice was associated with protection from tumor metastases".
In Abstract, line 6: the use of the term "related to" seemed an evasive why to describe a functional relationship.
The final part of the Abstract re patients who have severe adverse effects against PD-1 mAbs seemed out of step with the actual contents or implications of the study (-possibly an OTT effort to market the work to a translational audience?)
p3. line 5, typo: "has been shown"
Unclear here precisely what the authors mean by the term "clinical anti-tumour activity"
p3 para 2, line 3. The authors seem to imply that PD-1 effects have been limited to the tumor immunity field, which obviously is not the case
p5, para 3, typo " also showed decreased PD-1"
p6 para 1, line 2, typo "after being treated"

In Figure 1F labeling, the legend and text clarify that this shows over-expression rather than knockout, but this is not clear from looking at labeling of the figure itself. Also, I may have missed this, could not see data on the extent of over-expression after transfection?

The point about the phenotype of the knockouts is on the whole well made, but I was disappointed not to see any data from the double knockouts. What phenotype does this give?

p6, para 3, line 13: Would be more clearly rephrased along the lines of " Taken together, this offers the first data in support of....."

I would have found the tumor protection data more persuasive if some of the information from FigS4B was shown in the main paper and more extensively described. That is, we need to see number about numbers of metasteses, not just 'survival'. It would also be good to see more comprehensive analysis of T effector infiltrates.

One might have perhaps wished for a wider consideration of some more challenging tumor models in these studies, but, nevertheless, some very interesting findings.

Referee #2 (Comments on Novelty/Model System):

There is an ethical concern. In the paragraph: "SKAP55 deficient mice enhance DC-based vaccine for prevention of tumors in vivo", the authors analyse the ability of DC-based vaccination to prevent tumor growth in SKAp55-/- mice. Survival rates and tumor multiplicity are shown. Current Ethical Guideline recommends choosing endpoints that minimize pain and/or distress to the animals. The observation of the tumor multiplicity in the lung is an endpoint sufficient to assess the preventive anti-tumor efficacy of the vaccine and is a much more humane endpoint than survival. Survival curves should not be shown.

Referee #2 (Remarks):

The authors investigate the mechanisms underlying the expression of programmed death-1 (PD-1) during anti-tumor immunotherapy. The results show that the deficiency of ADAP or SKAP55 inhibits PD-1 expression and enhances the anti-tumor effectiveness of immunotherapy.

General comments:
Antibodies that block novel checkpoint molecules, including PD-1 and PD-L1, have demonstrated activity in multiple tumour types, in clinical and pre-clinical studies, therefore the study of the control of PD-1 expression is highly relevant for cancer research. On the whole, the data are very innovative and the results presented are very convincing. Nevertheless, the conclusions drawn are far away from the realistic conclusions suggested by the results. It would be advisable to rewrite the discussion more in the light of a mechanistic study rather than as if the authors had discovered a new

way of targeting PD-1.

Specific observations:
1. The manuscript should be carefully revised for the English.
2. There is an ethical concern. In the paragraph: "SKAP55 deficient mice enhance DC-based vaccine for prevention of tumors in vivo", the authors analyse the ability of DC-based vaccination to prevent tumor growth in SKAp55-/- mice. Survival rates and tumor multiplicity are shown. Current Ethical Guideline recommends choosing endpoints that minimize pain and/or distress to the animals. The observation of the tumor multiplicity in the lung is an endpoint sufficient to assess the preventive anti-tumor efficacy of the vaccine and is a much more humane endpoint than survival. Survival curves should not be shown.
3. There is also a major concern about the statistical analysis. Statistical significance was determined with two-tailed Student's t-test, the use of a non-parametric statistical test is strongly recommended.
4. In the discussion, the authors suggest a new usage of Cyclosporine A for tumour therapy. Nevertheless, in the present study only in vitro studies were done to support this hypothesis and concerns are raised by the fact that Cyclosporine A is a powerful immunosuppressant drug. It would be more appropriate to make this statement more cautious in view of the frequent observation of increased incidence of different types of tumors in individuals treated with cyclosporine.

Referee #3 (Comments on Novelty/Model System):

The high importance is given by the fact that anti-PD-1 is currently a novel and successful immune-therapy in cancer and by the clear effects seen in the authors mouse model system.
In my mind impact could be higher if they compare their results with the effect of CSA (see comments to authors. If CSA indeed down-regulates PD-1 in vivo it would certainly be of broad interest.

Referee #3 (Remarks):

In their manuscript Li and coworkers describe that PD-1 is upregulated on CD8 CTL when they are deficient of the ADAP-SKAP55 signaling module. This mechanism has implications for translational medicine as current cancer immunotherapy schemes use anti-PD-1 antibodies successfully to increase the effector-functions of anti-tumor CTL. At least in DC vaccine mouse model they demonstrate, that CTL deficient of ADAP-SKP55 have much higher tumor clearance potency than WT cells. Therefore the authors argue that this signaling module should be targeted for therapeutic reasons.
This is a well written manuscript on a timely subject which has the necessary mix of in vitro analyses, system manipulation and animal model data as well as the necessary technical quality. I have only a few comments.
1) The authors suggest that the ADAP-SKAP55 module should be targeted when/as side effects of anti-PD-1 treatment are severe. Usually this targeting would likely produce a drug which itself may have severe side effects unless the authors think of CSA which, in their assay systems, has a significant effect on PD-1 expression (figure 3D). Unfortunately they do not demonstrate statistical significance, although it looks like being significant, and they have not used CSA in their DC-vaccine mouse model system. Judging from the in vitro data, it would be interesting to compare the CSA effect with ADAP-SKAP55 deficiency. If the effect would be comparable, as could be expected from the in vitro data, this would be an important notion for the field and their study and CSA could be tested as a "poor man's" anti-PD-1 drug. In this case, the CSA effect should be included into the abstract and the keywords.
2) Without the proper controls (specific/nonspecific competitor probe) an EMSA as in figure 3C is not very meaningful and should be repeated. The indicated band could be nonspecific.

1st Revision - authors' response 27 January 2015

We thank the Editors and reviewers for providing constructive suggestions. We are glad that all reviewers thought our work is interesting and of potential importance. We also carefully considered their comments and performed substantially experiments. Please find the point-by-point responses to the Editors and reviewers' comments. The changes in the text and figures are labeled with red colour.

Editors' comments

Reviewer 1, S/he would like to see the data on the extent of over-expression of SKAP55 and on the double knock-outs. Finally, Reviewer 1 also suggests that the figure illustrating the data on tumour protection be made a main one and mentions that survival, as opposed to the numbers of metastases is not a good indicator.

We have followed the reviewer's suggestion and added new data about SKAP55 overexpression (Supplementary Fig. S1C) and the double KOs (Fig. 2F, Supplementary Fig. S2D, S3B, S6C). We also removed the survival curve from the revised manuscript.

Reviewer 2 similarly to Reviewer 1, suggests careful revision for English usage. … First, s/he suggests that the statistical analysis of your results should be redone with a better-suited approach. ... The other concern raised by Reviewer 2 is an ethical one with respect to animal experimentation;… In this respect the request by Reviewer 1 to provide better lung tumour data is especially fitting. I must also ask you to provide more details in the Materials and Methods section identifying the institutional and/orlicensing committee approving the experiments, also including any relevant details (like how many animals were used, of which gender, at what age, which strains, if genetically modified, on which background, housing details, etc). …Connected to this and the above-mentioned statistics issue, we are now requesting all Authors to complete an Author checklist (see below). ..

We have asked other scientists in our institutes to carefully check English usage.
We have redone statistical analysis withMann–Whitney U test.
Lung metastases (not survival curve) data were shown in Fig. 4A and Supplementary Fig. S5.
More details of the animal license and mouse information were added to the Materials and Methods section (page 18).
The Author checklist was completed as requested.

Reviewer 3 is also positive and raises one interesting point concerning cyclosporine A (CSA), which was also mentioned by Reviewer 2. Specifically, s/he would like to see further experimentation to establish significance and would like you, ideally, to compare the effect of CSA with ADAP-SAKP55 deficiency. I agree with him/her that this would significantly increase the relevance and impact of your manuscript and therefore encourage you to develop your study in this sense as far as realistically possible.

We thank the reviewer's helpful advice. We have checked the effect of CSA against tumor development in vivo. Similar to ADAP or SKAP55 deficient CD8$^+$ CTLs, injection of the in vitro CsA-pretreated CD8$^+$ CTLs enhanced the recipient mice against tumor growth in vivo (Fig. 7).

Referee #1 (Comments on Novelty/Model System):

There is no doubt that the area of PD-1 modulation of anti-tumor immunity is currently of intense and highly competitive interest with respect to translational immunology impacts. *This paper offers some new insights and may possibly offer some new therapeutic targets.* The reliance on the slightly 'easy win' of the B16 melanoma model possibly diminishes slightly ones enthusiasm for adequacy of the model system...One might have perhaps wished for a wider consideration of some more challenging tumor models in these studies, but, nevertheless, some very interesting findings.

Thanks for the reviewer's question. Except for B16 melanoma model, we also used the in vivo EG.7 lymphoma model or EG.7 cells in vitro to test how SKAP55 and ADAP regulated CTL lytic ability. Moreover, we used three in vivo Immunotherapy methods, including the protective DC vaccine and therapeutic DC vaccine models and the adoptive CTL transferring model.

Referee #1 (Remarks):
In this manuscript by Li and colleagues *a number of substantive new insights are offered* about the role of the SKAP-55/ADAP pathway in modulation of PD-1 expression and thus, the potential for modulatory effects on tumor killing. *The points are novel and in many respects, well evidenced by the data.*
I have a number of specific comments:
The Abstract could be considerably sharpened. The wording was sometimes ambiguous. For example, in line 5 where it is stated that "KO mice profoundly prevented tumor formation", this needs to be rephrased as something like "knockout of SKAP 55 or ADAP in mice was associated with protection from tumor metastases". In Abstract, line 6: the use of the term "related to" seemed an evasive why to describe a functional relationship.

We have followed the reviewer's suggestion to sharpen and rephrased the sentence the abstract.

The final part of the Abstract re patients who have severe adverse effects against PD-1 mAbs seemed out of step with the actual contents or implications of the study (-possibly an OTT effort to market the work to a translational audience?)

We have deleted this sentence in the abstract.

p3. line 5, typo: "has been shown"
Unclear here precisely what the authors mean by the term "clinical anti-tumour activity"

We have rephrased the sentence in the abstract.

New version of the Abstract:
PD-1 negatively regulates $CD8^+$ cytotoxic T lymphocytes (CTL) cytotoxicity and anti-tumor immunity. However, it is not fully understood how PD-1 expression on $CD8^+$ CTL is regulated during anti-tumor immunotherapy. In this study, we have identified that the ADAP-SKAP55 signaling module reduced $CD8^+$ CTL cytotoxicity and enhanced PD-1 expression in a Fyn-, Ca^{2+}-, and NFATc1-dependent manner. In DC vaccine based tumor prevention and therapeutic models, knockout of SKAP55 or ADAP showed a heightened protection from tumor formation or metastases in mice, and reduced PD-1 expression in $CD8^+$ effector cells. Interestingly, CTLA-4 levels and the percentages of tumor infiltrating $CD4^+Foxp3^+$ Tregs remained unchanged. Furthermore, adoptive transfer of SKAP55 deficient or ADAP deficient $CD8^+$ CTLs significantly blocked tumor growth and increased anti-tumor immunity. Pre-treatment of wild type $CD8^+$ CTLs with the NFATc1 inhibitor CsA could also downregulate PD-1 expression and enhance anti-tumor therapeutic efficacy. Together, we propose that targeting the unrecognized ADAP-SKAP55-NFATc1-PD-1 pathway might increase efficacy of anti-tumor immunotherapy.

p3, para 2, line 3. The authors seem to imply that PD-1 effects have been limited to the tumor immunity field, which obviously is not the case

We have rephrased it as "in response to various kinds of antigens such as tumor antigen" (page 3).

p5, para 3, typo " also showed decreased PD-1" p6, para 1, line 2, typo "after being treated"

We have changed to "also decreased PD-1" (page 5) and "after the treatment with"(page 6).

In Figure 1F labeling, the legend and text clarify that this shows over-expression rather than knockout, but this is not clear from looking at labeling of the figure itself. Also, I may have missed this, could not see data on the extent of over-expression after transfection?

SKAP55 or ADAP was overexpressed with GFP as fusion proteins. We have checked the expression levels of GFP-SKAP55 or GFP-ADAP by FACS after transfection (supplementary Figure S1C and S2C).

Figure S1C

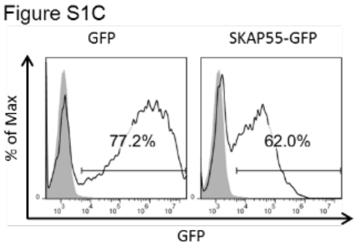

Figure S2C

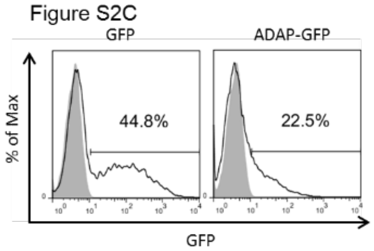

The point about the phenotype of the knockouts is on the whole well made, but I was disappointed not to see any data from the double knockouts. What phenotype does this give?

We and others previously reported that ADAP KO T cells show a loss of SKAP55 expression (Huang et al., 2005; Wang et al., 2007). The double knockout mice show similar phenotype as ADAP or SKAP55 knockout mice (Fig. 2F, Supplementary Fig. S2D, S3B and S6C). We apologize not providing this information in our previous manuscript, and now have added it in the Discussion (page 15, para 1).

Figure 2F

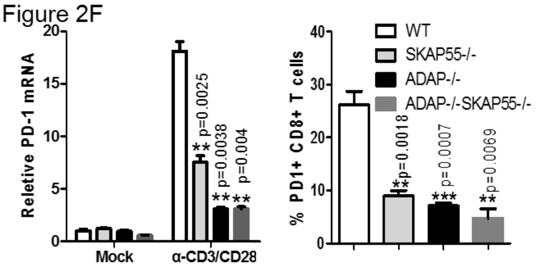

Figure S2D

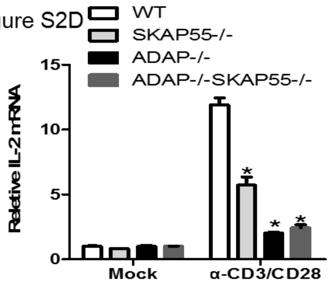

Figure S3B

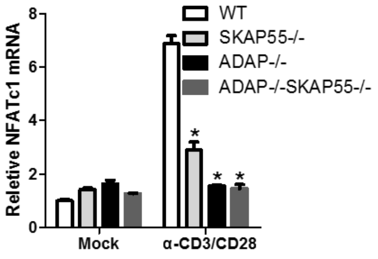

Figure S6C

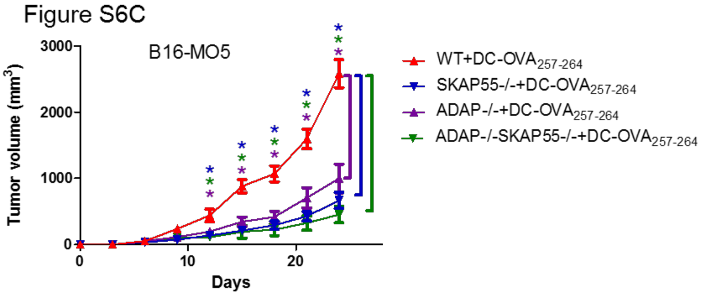

p6, para 3, line 13: Would be more clearly rephrased along the lines of " Taken together, this offers the first data in support of....."

We rewrote the sentence as suggested.

I would have found the tumor protection data more persuasive if some of the information from FigS4B was shown in the main paper and more extensively described. That is, we need to see numbers of metasteses, not just 'survival'. It would also be good to see more comprehensive analysis of T effector infiltrates.

We have taken the editor and the other reviewer's advice to remove Fig. S4B (the survival curve), and showed numbers of metasteses (Fig. 4A, tumors in lung). Additional analysis including the expression of CD25, CD69, IFN-gamma, Perforin was added in Fig. S4B.

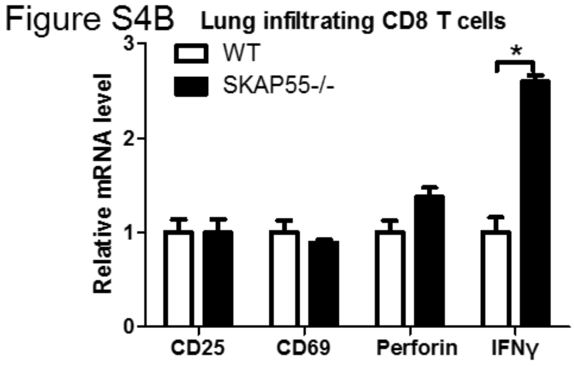

Referee #2 (Comments on Novelty/Model System):
There is an ethical concern. In the paragraph: "SKAP55 deficient mice enhance DC-based vaccine for prevention of tumors in vivo", the authors analyse the ability of DC-based vaccination to prevent tumor growth in SKAp55-/- mice. Survival rates and tumor multiplicity are shown. Current Ethical Guideline recommends choosing endpoints that minimize pain and/or distress to the animals. The observation of the tumor multiplicity in the lung is an endpoint sufficient to assess the preventive anti-tumor efficacy of the vaccine and is a much more humane endpoint than survival. Survival curves should not be shown.

Referee #2 (Remarks):
The authors investigate the mechanisms underlying the expression of programmed death-1 (PD-1) during anti-tumor immunotherapy. The results show that the deficiency of ADAP or SKAP55 inhibits PD-1 expression and enhances the anti-tumor effectiveness of immunotherapy.

General comments:
Antibodies that block novel checkpoint molecules, including PD-1 and PD-L1, have demonstrated activity in multiple tumour types, in clinical and pre-clinical studies, therefore *the study of the control of PD-1 expression is highly relevant for cancer research. On the whole, the data are very innovative and the results presented are very convincing.* Nevertheless, the conclusions drawn are far away from the realistic conclusions suggested by the results. It would be advisable to rewrite the discussion more in the light of a mechanistic study rather than as if the authors had discovered a new way of targeting PD-1.

We have rewritten the discussion part as suggested (page 15/16).

Specific observations:
1. The manuscript should be carefully revised for the English.

We have carefully revised our manuscript.

2. There is an ethical concern. … The observation of the tumor multiplicity in the lung is an endpoint sufficient to assess the preventive anti-tumor efficacy of the vaccine and is a much more humane endpoint than survival. Survival curves should not be shown.

We thank the reviewer's advice and have removed the survival curve.

3. There is also a major concern about the statistical analysis. Statistical significance was determined with two-tailed Student's t-test, the use of a non-parametric statistical test is strongly recommended.

We have re-done statistical analysis with Mann–Whitney U-test.

4. In the discussion, the authors suggest a new usage of Cyclosporine A for tumour therapy. Nevertheless, in the present study only in vitro studies were done to support this hypothesis and concerns are raised by the fact that Cyclosporine A is a powerful immunosuppressant drug. It would be more appropriate to make this statement more cautious in view of the frequent observation of increased incidence of different types of tumors in individuals treated with cyclosporine.

We have carefully titrated the concentration of CsA that was used to treat WT CTLs in vitro. Then the CsA-treated CTLs were adoptively transferred into the recipient mice that were previously injected with B16 to form tumors under skin. The CsA-treated CTLs could enhance lytic ability to clear tumor effectively in vivo (Fig. 7).

We agreed with the reviewer that CsA is an immunosuppressive reagent and discussed this issue in page 16 "In contrast, we observed that the in vitro CsA pre-treated CD8$^+$ CTLs reduced PD-1 expression, and injection of the in vitro CsA pre-treated CD8$^+$ CTLs could enhance the recipient mice against tumor growth. Other studies suggest that CsA inhibits tumor growth due to a blockage of cell cycle and the induction of necrosis, supporting the usage of CsA in anti-tumor therapy (Pyrzynska et al, 2002; Werneck et al, 2012). We propose that the in vitro CsA pre-treatment of CD8$^+$ CTLs and the restricted local injection of low dose CsA only in solid tumors might limit the immunosuppressive role of CsA. Thus, it is possible to consider a new usage of CsA for anti-tumor therapy by targeting PD-1 expression in CD8$^+$ T cells or by targeting tumor itself".

Figure 7

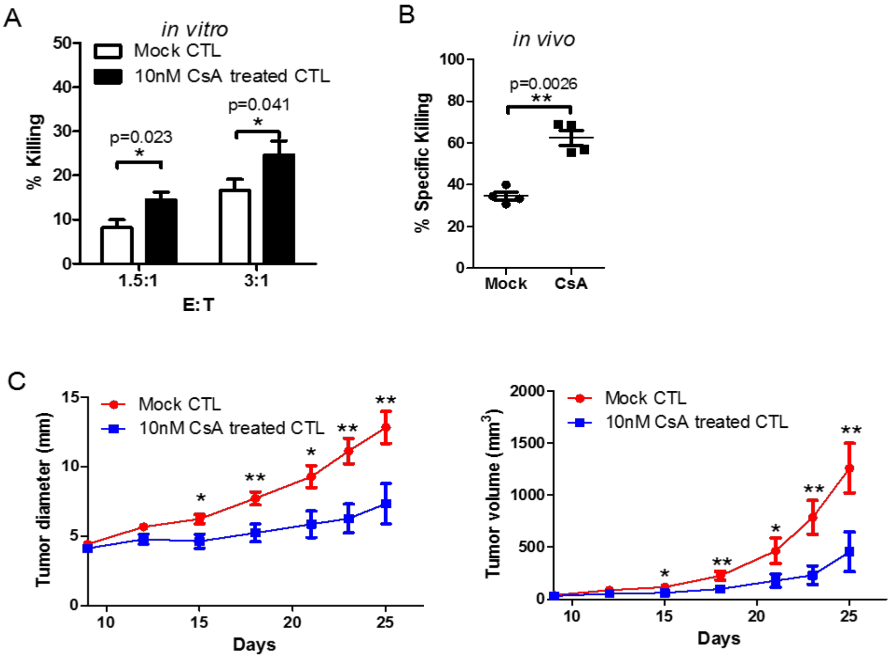

Referee #3 (Comments on Novelty/Model System):
The high importance is given by the fact that anti-PD-1 is currently a novel and successful immune-therapy in cancer and by the clear effects seen in the authors mouse model system.
In my mind impact could be higher <u>if they compare their results with the effect of CSA</u> (see comments to authors. If CSA indeed down-regulates PD-1 in vivo it would certainly be of broad interest.

Referee #3 (Remarks):
In their manuscript Li and coworkers describe that PD-1 is upregulated on CD8 CTL when they are deficient of the ADAP-SKAP55 signaling module. This mechanism has implications for translational medicine as current cancer immunotherapy schemes use anti-PD-1 antibodies

successfully to increase the effector-functions of anti-tumor CTL. At least in DC vaccine mouse model they demonstrate, that CTL deficient of ADAP-SKP55 have much higher tumor clearance potency than WT cells. Therefore the authors argue that this signaling module should be targeted for therapeutic reasons. *This is a well written manuscript on a timely subject which has the necessary mix of in vitro analyses, system manipulation and animal model data as well as the necessary technical quality.* I have only a few comments.
1) The authors suggest that the ADAP-SKAP55 module should be targeted when/as side effects of anti-PD-1 treatment are severe. Usually this targeting would likely produce a drug which itself may have severe side effects unless the authors think of CSA which, in their assay systems, has a significant effect on PD-1 expression (figure 3D). Unfortunately they do not demonstrate statistical significance, although it looks like being significant, and they have not used CSA in their DC-vaccine mouse model system. Judging from the in vitro data, it would be interesting to compare the CSA effect with ADAP-SKAP55 deficiency. If the effect would be comparable, as could be expected from the in vitro data, this would be an important notion for the field and their study and CSA could be tested as a "poor man's" anti-PD-1 drug. In this case, the CSA effect should be included into the abstract and the keywords.

We have demonstrated statistical significance in Figure 3D.
CsA was used to pre-treat WT CTLs during priming stage in vitro. Then the CsA-treated CTLs were adoptively transferred into mice that had been injected with B16 to form tumors under skin. The CsA-treated CTLs could clear tumor more effectively in vivo (Fig. 7C). We have discussed the potential usage of CsA for tumor therapy. Followed the reviewer's advice, we added CsA effect in the abstract and the keywords.

2) Without the proper controls (specific/nonspecific competitor probe) an EMSA as in figure 3C is not very meaningful and should be repeated. The indicated band could be nonspecific.

We have included the specific/nonspecific competitor probes as the controls in Figure 3C. In the DNA binding competition assays, we used the unlabeled probe (Comp. N1) or the mutant probe that contains the same sequence except for carrying a mutated NFAT binding site (Comp. mutN1). We *observed that Comp. N1 abrogated the formation of the probe-NFAT complex, while Comp. mutN1 failed to achieve this competition, indicating specificity of the NFAT binding probe. I addition, the*

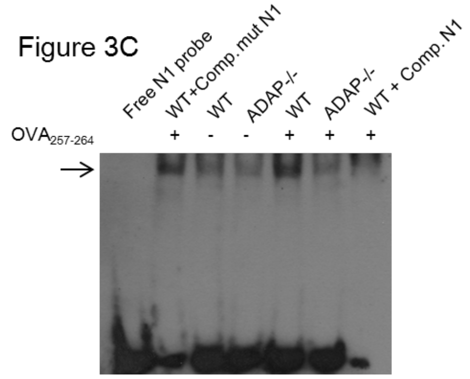

specificity of this probe has been described by Oestreich et al (Oestreich et al., 2008).

We believe that we have adequately addressed all of the criticisms raised by the reviewers and that our manuscript has been substantially improved. We thus hope that our work is now suitable for publication in EMBO Molecular Medicine.

References:

Huang, Y., Norton, D.D., Precht, P., Martindale, J.L., Burkhardt, J.K., and Wange, R.L. (2005). Deficiency of ADAP/Fyb/SLAP-130 destabilizes SKAP55 in Jurkat T cells. The Journal of biological chemistry *280*, 23576-23583.
Oestreich, K.J., Yoon, H., Ahmed, R., and Boss, J.M. (2008). NFATc1 regulates PD-1 expression upon T cell activation. Journal of immunology *181*, 4832-4839.
Wang, H., Liu, H., Lu, Y., Lovatt, M., Wei, B., and Rudd, C.E. (2007). Functional defects of SKAP-55-deficient T cells identify a regulatory role for the adaptor in LFA-1 adhesion. Molecular and cellular biology *27*, 6863-6875.

2nd Editorial Decision 13 February 2015

Thank you for the submission of your revised manuscript to EMBO Molecular Medicine.

We have now received the enclosed reports from the referees that were asked to re-assess it. As you will see the reviewers are now globally supportive and I am pleased to inform you that we will be able to accept your manuscript pending the following final amendments:

1) Reviewer 3 suggests that you perhaps comment in the discussion that pre-treatment of T cells with CsA before adoptive transfer might be a possible indication. I agree that this would be interesting/useful.
2) Please provide a new supplementary information file without red lettering.
3) We are now encouraging the publication of source data, particularly for electrophoretic gels and blots, with the aim of making primary data more accessible and transparent to the reader. Would you be willing to provide a PDF file per figure that contains the original, uncropped and unprocessed scans of all or at least the key gels used in the manuscript? The PDF files should be labeled with the appropriate figure/panel number, and should have molecular weight markers; further annotation may be useful but is not essential. The PDF files will be published online with the article as supplementary "Source Data" files. If you have any questions regarding this just contact me.
4) Every published paper now includes a 'Synopsis' to further enhance discoverability. Synopses are displayed on the journal webpage and are freely accessible to all readers. They include a short standfirst (to be written by the editor) as well as 2-5 one sentence bullet points that summarise the paper (to be written by the author). Please provide the short list of bullet points that summarise the key NEW findings. The bullet points should be designed to be complementary to the abstract - i.e. not repeat the same text. We encourage inclusion of key acronyms and quantitative information. Please use the passive voice. Please attach these in a separate file or send them by email, we will incorporate them accordingly.

I look forward to reading a new revised version of your manuscript as soon as possible and in any case, within two weeks

***** Reviewer's comments *****

Referee #1 (Comments on Novelty/Model System):

Much sharper now than earlier version

Referee #2 (Remarks):

The authors adequately addressed all my criticisms and improved the quality of the manuscript

Referee #3 (Comments on Novelty/Model System):

I have read the paper again and I think the authors have made a real efforts to improve the manuscript. They have addressed my comments/criticism adequately. With the additional CsA experiments the whole story is more complete and nicely links the mouse experiments to translational medicine. I am not sure whether physicians will treat their tumor patients with cyclosporine in the future, however, elucidating what the drug is doing is certainly important for the field. On the other hand, pretreatment of T cells with CsA before adoptive transfer to tumor patients may be an indication for CsA and a consequence of their study.

Referee #3 (Remarks):

I have no further comments to the authors. On a last note, however, the authors could mention in the discussion, that pretreatment of T cells with CsA before adoptive transfer to tumor patients could be an indication for this drug.
