# Supplementary figures and images for: ADAP and SKAP55 deficiency suppresses PD-1 expression in CD8+ cytotoxic T lymphocytes for enhanced anti-tumor immunotherapy
Source: EMBO Mol Med. 2015 Apr 7;7(6):754–69. doi: 10.15252/emmm.201404578 (PMC4459816; doi:10.15252/emmm.201404578)

Source data of Figure 3A

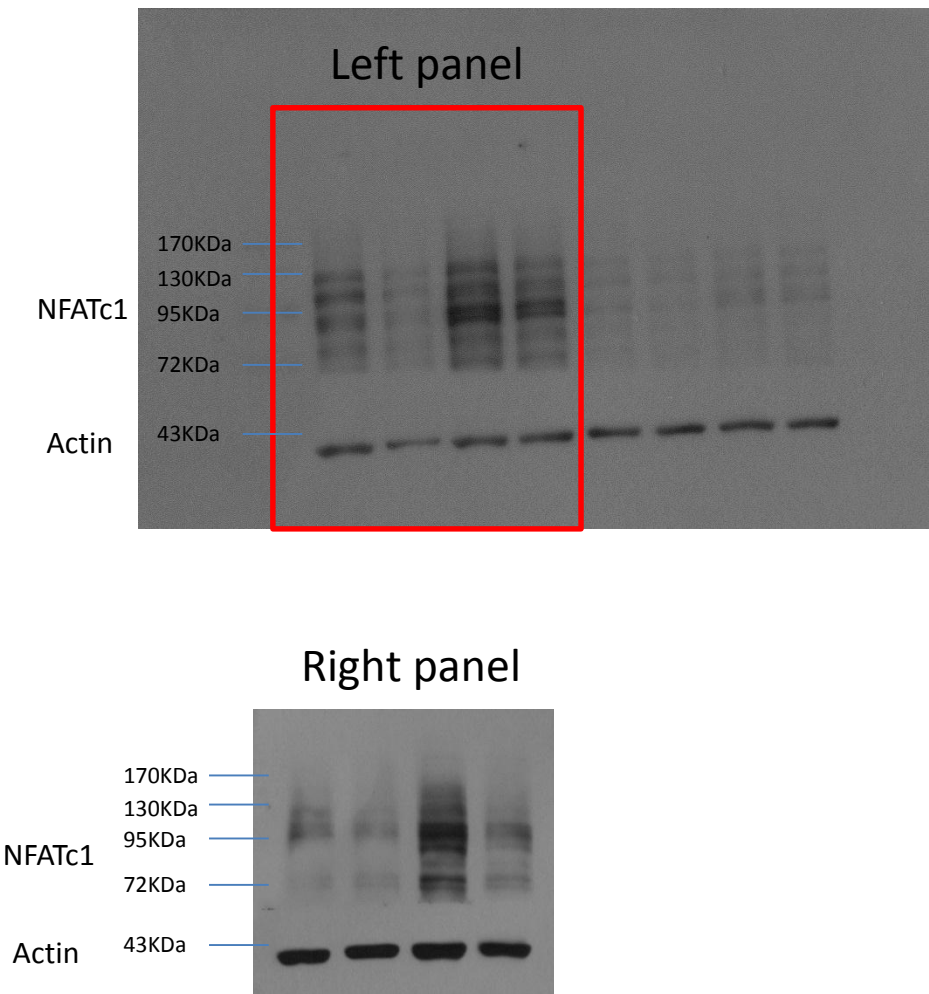

Supplement: Supplementary file 3 [file emmm0007-0754-sd3.pdf]
